# Assessment of genetic conservation units of an endangered glacial relict insular species, *Amentotaxus formosana*, based on fine-scale genetic structures of multiple fragmented mountainous populations in Taiwan

**DOI:** 10.3389/fpls.2024.1512914

**Published:** 2025-01-16

**Authors:** Ya-Zhu Ko, Huie-Chuan Shih, Chin-Shang Ho, Chaur-Tzuhn Chen, Tsai-Wen Hsu, Meng-Shin Shiao, Yu-Chung Chiang

**Affiliations:** ^1^ Department of Biological Sciences, National Sun Yat-sen University, Kaohsiung, Taiwan; ^2^ Department of Nursing, Meiho University, Pingtung, Taiwan; ^3^ Graduate Institute of Bioresources, National Pingtung University of Science and Technology, Pingtung, Taiwan; ^4^ Department of Forestry, National Pingtung University of Science and Technology, Pingtung, Taiwan; ^5^ Taiwan Biodiversity Research Institute, Nantou, Taiwan; ^6^ Office of Research, Academic Affairs and Innovations, Faculty of Medicine Ramathibodi Hospital, Mahidol University, Bangkok, Thailand; ^7^ Department of Biomedical Science and Environment Biology, Kaohsiung Medical University, Kaohsiung, Taiwan

**Keywords:** *Amentotaxus formosana*, population genetic structure, demographic dynamics, management units, microsatellites

## Abstract

Insular species are usually endemic and prone to long-term population reduction, low genetic diversity, and inbreeding depression, which results in difficulties in species conservation. The situation is even more challenging for the glacial relict species whose habitats are usually fragmented in the mountainous regions. *Amentotaxus formosana* is an endangered and endemic relict tree species in Taiwan. It is typically found scattered across different mountain regions, leading to fragmented and discontinuous populations in various habitats. Consequently, reproductive isolation may lead to deep and cryptic population structures between and within populations. To test this hypothesis and assess the most effective conservation units for the species, comprehensive genetic analyses were conducted using multiple microsatellite loci to uncover fine-scale population structures in three mountainous regions. In this study, a substantial sample of over 600 individuals, predominantly middle-aged, was collected, representing a majority of the existing individuals in the Chachayalaishan and Dawu habitats. Population genetic structure analyses were conducted using three complementary Bayesian clustering approaches (STRUCTURE, InStruct, and GENELAND) to ensure robust identification of distinct genetic clusters in three key habitats (CHA, DAWU, and DL). Results indicated low genetic diversity, distinct genetic differentiation, and severe inbreeding within fragmented populations. Additionally, demographic analysis suggested a substantial downfall in effective population sizes and limited gene flow within and between populations. Based on these findings, we recommend several management strategies to ensure the effective preservation of *A. formosana*: (1) establishing genetic conservation units corresponding to distinct genetic clusters in the CHA (CG-8-1 to CG-8-8), DAWU (DG-15-1 to DG-15-15), and DL (LG-6-1 to LG-6-6) populations; (2) implementing interpopulation cross-breeding programs to enhance genetic diversity; (3) promoting habitat restoration efforts to establish connectivity among fragmented populations; and (4) implementing vegetative propagation of selected trees for *ex-situ* conservation, along with establishing conservation nurseries and seed production areas. These localized conservation approaches, combined with the comprehensive genetic insights provided by this study, serve as crucial directives for the genetic monitoring, policy formulation, and sustainable conservation of *A. formosana*.

## 1 Introduction

Insular species encounter numerous threats that complicate conservation efforts. They are vulnerable to demographic fluctuations, prolonged population declines, and reductions in effective population size, while also being sensitive to microenvironmental changes. These challenges lead to decreased genetic diversity, inbreeding depression, and the potential for species extinction (Frankham, 1997; Woolfit and Bromham, 2005). In Taiwan’s mountainous island environment, climate change may include alterations in temperature and precipitation patterns, increased typhoon intensity, and rising frequency of regional extreme climate events, all of which can have significant impacts on the small and isolated habitats of insular populations. As a result, there is an urgent need for robust conservation strategies, including the identification of genetic conservation units, particularly for endemic and endangered insular species.

Identifying these genetic conservation units is crucial, as they form the basis for enhancing protective measures. These units serve as indicators for management and monitoring and play a vital role in the legal establishment of species and habitat protection (Allendorf and Luikart, 2007). The success in identifying and managing genetic conservation units of a species increases its capacity to adapt to future environmental change and refrain from extinction. Different concepts have been proposed for managing genetic conservation units: species identification, Evolutionary Significant Units (ESUs), and Management Units (MUs) (Ryder, 1986; Moritz, 1994). ESUs and MUs both consider conservation at the population level, with ESUs focusing on long-term historical population structures and MUs prioritizing current population structures for short-term management (Allendorf and Luikart, 2007). These management approaches guide our investigation of population structures to establish effective conservation strategies.

Taiwan is considered one of the hotspot habitats for glacial relict insular plant species due to its mountainous landscape, which has become the refuge for species that survived climate change during the during the Pleistocene, including the Last Glacial Maximum (Chou et al., 2011). Among the diverse glacial relict plants in Taiwan, several species such as *Taiwania cryptomerioides* Hayata, *Castanopsis carlesii* Hayata, *Cephalotaxus wilsoniana* Hayata, *Cycas taitungensis* C. F. Shen, K. D. Hill, C. H. Tsou and C. J. Chen, *Keteleeria davidiana* (Franch.) Beissner var. *formosana* Hayata, *Phoenix hanceana* Naudin and *Amentotaxus formosana* H. L. Li have been extensively studied. These glacial relict endemic insular species typically have small populations and low genetic diversity due to fragmented habitats in the mountains. Their limited pollination and seed dispersal abilities further increase inbreeding risk and reduce genetic exchange between populations. These characteristics together make them vulnerable to extinction without proper management and conservation strategies (Huang et al., 2001, 2004; Cheng et al., 2005; Chiang et al., 2009; Chou et al., 2011; Ge et al., 2014).

*Amentotaxus*, a particularly vulnerable genus, consists of five or six ancient gymnosperm species, depending on the classification system, was once widely distributed in the Northern Hemisphere (Farjon, 2001, 2010; Follieri, 2010; Yang et al., 2017). Fossil evidence shows that the genus thrived in pre-Quaternary Cenozoic periods, ranging from the Paleocene to the Upper Miocene, and was still present in the Middle Pleistocene in Peperino deposits of Europe, such as Italy, about 450,000 years ago. However, its distribution began to decrease dramatically during the Pleistocene, likely due to climatic oscillations associated with glacial and interglacial periods and continued to decline into the Holocene as warming trends forced population retreats to fragmented, specialized habitats across Asia (Follieri, 2010). Consequently, small populations are now preserved in a scattered pattern in the understory of moist submontane and montane semi-deciduous or evergreen forests across southern and central China, Taiwan, the eastern Himalayas, Vietnam, and Laos (Ferguson et al., 1978; Yang, 1993; Yang et al., 2008; Farjon, 2010). With specific requirements of habitat environments and slow growth rates, the *Amentotaxus* species face the risk of extinction due to significant habitat loss in recent years (Li et al., 2003; Hiep et al., 2004; Yang et al., 2008; Ruan et al., 2019). Currently, most *Amentotaxus* species are considered vulnerable or endangered in their endemic habitats and are protected under different government acts (Cheng, 1978; Li et al., 2003; Ban et al., 2007; Nguyen et al., 2013; Editorial Committee of the Red List of Taiwan Plants, 2017; Thomas et al., 2017; Chiang, 2023; Gao, 2023).

Among these, *A. formosana* H. L. Li is an endangered endemic insular species in Taiwan. The populations were found in the southernmost part of the tailing area of the Central Mountain Range in Taiwan, including Dalili Mountain, Dahan Mountain, Guzilun Mountain, Lilung Mountain, Chingshuiying Historial Trail, Dawu Taiwan Amentotaxus Nature Reserve, and Chachayalaishan Major Wildlife Habitat regions (Zhang, 1992; Yang et al., 2008; Yeh et al., 2012). The northernmost distribution of *A. formosana* is on the west side of Dalili Mountain’s eastern ridge 1256 peak and the west side of Tuyayuan Mountain’s ridge, while the southernmost distribution is on Lilung Mountain (Yang et al., 2008). *Amentotaxus formosana* is currently under legal protection in Taiwan due to its fragmented distribution, small population size, and poor sexual reproduction, which means any change or destruction of the habitat can easily threaten its survival and lead to extinction. In 1988, the Council of Agriculture granted official protection of *A. formosana* under the Cultural Heritage Preservation Act, which recognized it as a rare and endangered species. The Council of Agriculture has designated two locations, Dawu Taiwan *Amentotaxus* Nature Reserve and Chachayalaishan Major Wildlife Habitat, to preserve the species due to its endangered situation.

Extensive ecological surveys of *A. formosana*’s habitat have been conducted (Wu, 1992; Yeh et al., 1992; Zhang, 1992; Yang, 1993; Yang et al., 2008; Pingtung Forestry Management Office, 2009; Ho et al., 2012c). Notably, comprehensive assessments of *A. formosana* populations have been conducted, with each individual plant in the Dawu Taiwan *Amentotaxus* Nature Reserve and Chachayalaishan Major Wildlife Habitat being mapped with specific coordinates, along with detailed records of tree height, Diameter at Breast Height (DBH), and forest health indexes (Pingtung Forestry Management Office, 2009; Ho et al., 2012c). Despite thorough surveys and protective measures, *A. formosana* still lacks targeted genetic management strategies critical for its long-term survival. This situation underscores the urgent need for conservation initiatives that effectively integrate both ecological and genetic approaches.

While previous studies have examined small subsets of *A. formosana* populations in the Dawu Taiwan *Amentotaxus* Nature Reserve and Chachayalaishan Major Wildlife Habitat (Wang et al., 1996; Ge et al., 2005; Ho et al., 2012b), these studies were limited by small sample sizes and excluded populations from other habitats. Therefore, a more comprehensive investigation of island populations using high-resolution genetic markers is necessary to establish effective conservation strategies. This study comprehensively investigated the genetic diversity and population structures of *A. formosana* in Taiwan with ~700 individuals collected from three main habitats. We hypothesized that the species may possess cryptic genetic structures and, thus, multiple management units have to be considered for future conservation strategy. To achieve better assessment and identification of genetic conservation units, we aim to (i) identify the genetic variability of *A. formosana* populations across different habitats; (ii) explore the fine-scale spatial genetic structures within and across different populations from three habitats; (iii) evaluate the historical demography of the species and gene flow between populations, and (iv) define genetic conservation units based on the identified genetic structures in populations. This information will provide precious information in future genetic monitoring and effective conservation measures for endangered endemic insular relict species. Most importantly, this may serve as the template for other endemic insular species conservation.

## 2 Materials and methods

### 2.1 Sample collection

**Figure 1:**
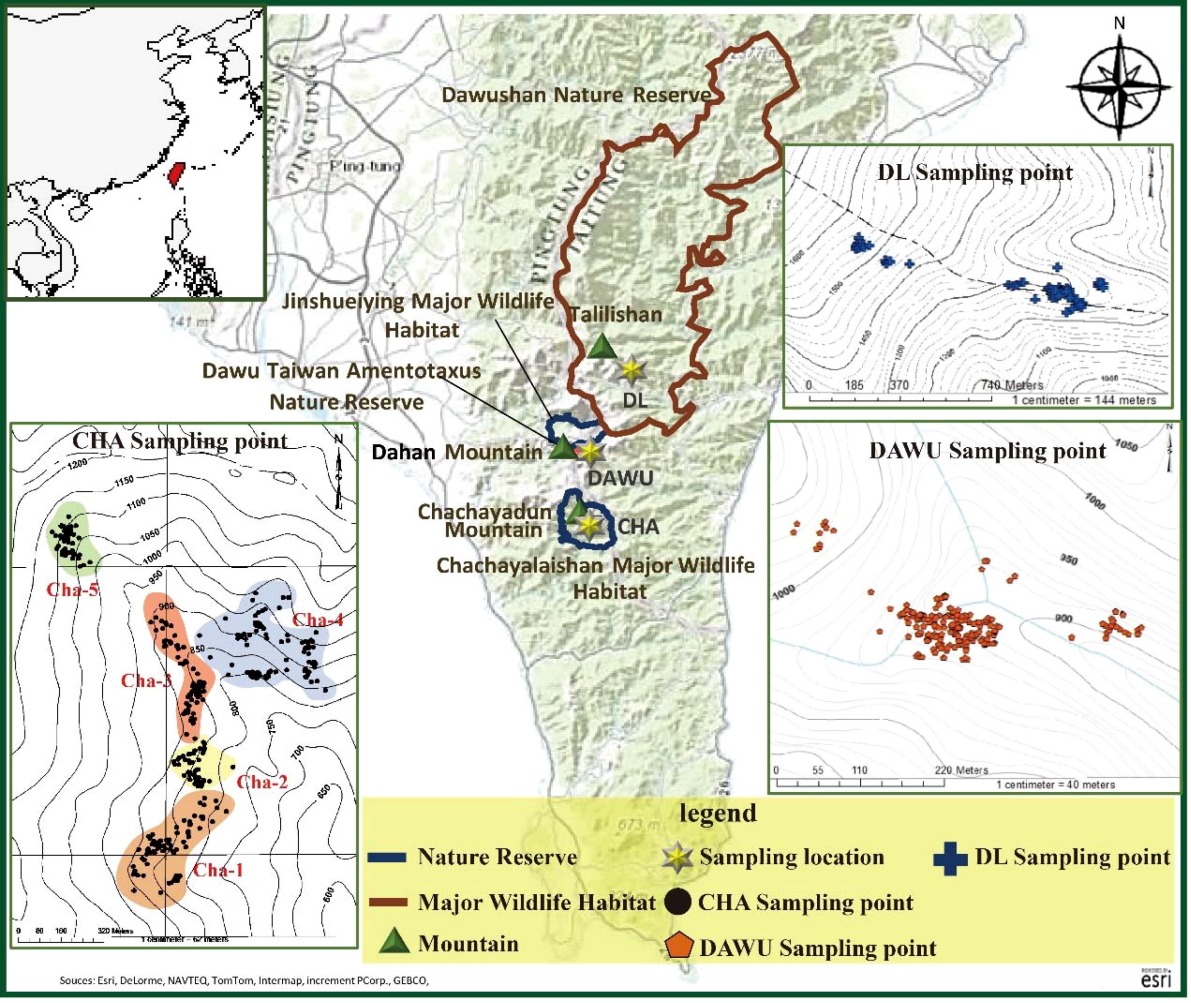
Map of sampling locations of *A. formosana*, including the three populations of Chachayalaishan Major Wildlife Habitat regions (CHA), Dawu Taiwan *Amentotaxus* Nature Reserve (DAWU) and Dalili (DL).

Samples from three primary habitats were collected: the Chachayalaishan Major Wildlife Habitat, the Dawu Taiwan *Amentotaxus* Nature Reserve, and the population at the Dalili Mountain’s eastern ridge 1256 peak (**Figure 1**). Leaf samples were collected from individual trees across the study area. To ensure systematic sampling that captured environmental variation, we established sampling plots of 40 × 25 m² (0.1 ha) across different altitudes and slopes, considering the distribution of *A. formosana* vegetation (Ho et al., 2012c). Within each plot, we sampled all *A. formosana* individuals present.

In total, we collected leaves from 678 individual trees (**Supplementary Table S1**). In the Dawu Taiwan *Amentotaxus* Nature Reserve, 228 samples were collected, hereinafter referred to as the “DAWU” populations. In the Dalili Mountain’s eastern ridge 1256 peak, 67 samples were collected and were referred to as the “DL” population (**Figure 1**; **Supplementary Table S1**). In the Chachayalaishan Major Wildlife Habitat, 383 samples from five distinct sampling plots were collected and referred to as the CHA population, including five distinct sampling plots: 82 (Cha-1), 55 (Cha-2), 91 (Cha-3), 96 (Cha-4), and 59 (Cha-5) samples were collected from each area (**Figure 1**; **Supplementary Table S1**). All samples contained detailed information, including altitude, latitude and longitude coordinates, tree height, ground diameter, health level, and Diameter at Breast Height (DBH) of the plants. The health status assessment followed the methodologies developed by Ho et al. (2012c) and the Forestry Bureau Pingtung Forest District Management Office (2009) (Pingtung Forestry Management Office, 2009; Ho et al., 2012c). Fresh leaf tissues from each sample were dried using a silica gel desiccant for genomic DNA extraction.

### 2.2 DNA-extraction and microsatellite amplification

Genomic DNA was extracted from dried leaf samples of *A. formosana* using a modified CTAB method (Doyle and Doyle, 1987). The extracted DNA was dissolved in 200 μl of Tris-EDTA (TE) buffer and stored at -20 °C. The quality of all extracted genomic DNA was assessed using 1% agarose gel electrophoresis with the Lambda marker (200 μg/mL, Promega, Madison, Wisconsin, USA) as the standard. DNA was diluted to a solution of 10ng/μl using TE buffer, which was used as the template DNA solution for subsequent experiments. Fifteen pairs of polymorphic microsatellite primers, previously described by Ho et al. (2012a) were amplified and genotyped for all samples of *A. formosana* genomic DNA (**Supplementary Table S2**) (Ho et al., 2012a). PCR reactions were performed using the Labnet MultiGene 96-well Gradient Thermal Cycler (Labnet, Edison, New Jersey, USA) with initial denaturation of 94°C for 2 min, followed by 35-40 cycles of 45s at 94°C, 1 min at 52-60°C, 30s at 72°C, and a final extension of 7 min at 72°C. The PCR products were separated and assessed using 10% polyacrylamide gel electrophoresis (mixed of 30% acrylamide, 5X TBE buffer, 10% Ammonium peroxydisulfate, and Tetramethyl ethylenediamine) performed in 1×TBE as the electrophoresis buffer at 70V for 16 h. The gels were then stained with ethidium bromide and visualized under UV light exposure. The alleles’ patterns and sizes were recorded digitally using Quantity One ver. 4.62 (Bio-Rad Laboratories, Hercules, California, USA). The genotyping of microsatellite amplification products was conducted following the methodology described by Mantiquilla et al. (2022) (Mantiquilla et al., 2022).

### 2.3 Data statistical analyses

#### 2.3.1 Detection of outlier loci and genetic diversity

LOSITAN (Antao et al., 2008) software was used to evaluate whether a microsatellite locus is under selection based on the *F_ST_* outlier approach. LOSITAN analyses were performed using a mean neutral *F_ST_* in an infinite allele model with 1,000,000 simulations and a subsample of 50. We applied the False Discovery Rate (FDR) correction with a p-value threshold of 0.01 using the main algorithm FDIST2 in LOSITAN (Beaumont and Nichols, 1996; Burford Reiskind et al., 2018). Locating outliers involved identifying loci with a distinct differentiation pattern (*F_ST_*) that deviated from the null distribution in the simulation (Antao et al., 2008). A locus with higher gene frequency in different populations may indicate positive selection, leading to higher genetic differentiation and exhibiting upward outlier *F_ST_* values. Conversely, a locus under balancing selection shows lower genetic differentiation and exhibits downward outlier *F_ST_* values. Once outliers are identified using the LOSITAN, they are removed from downstream analyses to obtain estimates of neutral population genetic structure and population demography.

The polymorphism information content (PIC) was calculated for each primer to estimate the degree of polymorphism at the microsatellite loci using Cervus v3.0.7 (Kalinowski et al., 2007). Values of 0.5 and 0.25 are recommended as criteria to categorize the polymorphic information of a locus as high (PIC > 0.5), moderate (0.25-0.5), or low (< 0.25) (Botstein et al., 1980).

GenAlEx v6.5 (Smouse et al., 2017) was used to calculate the observed heterozygosity (*H_O_*) and expected heterozygosity (*H_E_*), as well as the number of alleles (*Na*) and the effective number of alleles (*Ne*) for each sample location. To test for deviations from the Hardy-Weinberg equilibrium in each population across all loci based on gene frequency, the chi-square test (*χ^2^*) was employed using the same software, GenAlEx v6.5 (Smouse et al., 2017).

#### 2.3.2 Genetic differentiation

The hierarchical analysis of molecular variance (AMOVA) via the Arlequin v3.5 programs (Excoffier and Lischer, 2010) was employed to examine variance partitioning into components derived from different levels and assess the *F*-statistics significance using 9999 permutations. AMOVA was computed on two different levels to detect and perform *F* statistical analysis. It calculates fixation indices at different levels, which evaluate *F_ST_* (between populations), *F_CT_* (between groups), *F_SC_* (within populations of groups), as well as *F_IS_* and *F_IT_*. It also tests the 95% confidence interval to assess its significance.

#### 2.3.3 Cluster analysis

To accurately explore the genetic structure pattern and delineate the genotype clusters, three Bayesian assignment test methods are utilized to study the genetic structure of three wild populations of *A. formosana*. The tests were conducted at two levels. First, all sampling sites are integrated for analysis to detect the cryptic genetic groups of *A. formosana* in Taiwan. Second, a subset of each *A. formosana* population is independently analyzed to gain better insight into the fine-scale cryptic genetic groups within each area. A series of special codes representing each genotype division result is used to explain the results of genotype division at different levels by three different clustering programs (STRUCTURE, InStruct, and GENELAND).

STRUCTURE v2.3.4 (Pritchard et al., 2000) to explore the possibility of cryptic structure assuming admixture, correlated allele frequencies, and co-ancestry. We calculated the posterior probabilities of 20 independent MCMC chains for each K value (1–20). The chains were run with 10^5^ burn-in periods followed by 10^6^ Monte Carlo Markov Chain (MCMC) replicates collected after burn-in to obtain accurate parameter estimates. To verify the convergence of the chain and the consistency across each prior K run, we performed twenty independent runs of the Markov chain. The output file generated by the STRUCTURE analysis was evaluated and visualized using the STRUCTURE HARVESTER program to assess likelihood values across multiple k values and iterations (Earl and VonHoldt, 2012). To detect the highest hierarchical level of structure present in the data, we utilized the *ad hoc* summary statistic ΔK, as proposed by Evanno et al. (2005) (Evanno et al., 2005). The most suitable K value was selected and CLUMPP was used to align multiple replicates of the chosen K cluster in the STRUCTURE analysis results to address multimodality problems (Jakobsson and Rosenberg, 2007). Finally, the POPHELPER v2.3.1 R package created bar plots to present the STRUCTURE output (Francis, 2017).

The STRUCTURE program assumes a fully outcrossing situation in the sample area. If applied to a population of self-crossing or inbreeding, it may result in pseudo-subpopulation structures and misjudgment of the results (Falush et al., 2003). Therefore, we also utilized the InStruct (Gao et al., 2007) analysis to validate the clustering results. InStruct uses the Bayesian clustering method to perform clustering work through MCMC operations. Unlike STRUCTURE, InStruct does not assume Hardy-Weinberg equilibrium and uses multiple genetic markers to estimate allele frequency. It provides inbreeding or selfing values and eliminates false subpopulation structures (Gao et al., 2007). The InStruct conditions are as follows: 10^5^ burn-in periods followed by 10^6^ MCMC replicates collected after burn-in. For simulation clustering, use a number K=1-20 and run 20 chains for each set of conditions to select the most suitable K value. Visualize the results using the POPHELPER v2.3.1 R package (Francis, 2017) to display the clustering results. The optimal number of clusters in InStruct was identified using the highest ΔK method proposed by Evanno et al. (2005) and the lowest value of the deviance information criterion (DIC) as recommended by Gao et al. (2011) (Evanno et al., 2005; Gao et al., 2011).

GENELAND v6.501 was performed in R v4.1.0, which integrated multilocus genetic data with geographic spatial information to ascertain a realistic depiction of the subpopulation’s geographic structure (Guillot et al., 2005). GENELAND analyses were executed using the correlated model of allele frequencies. The Markov Chain Monte Carlo (MCMC) runs to ensure the convergence of the chain performed ten independent runs with 100,0000 iterations. The thinning and the burn-in period were set to 100 and 100,000. The maximum number of populations (K) was set to 30. The Poisson process maximum rate and maximum number of nuclei in the Poisson-Voronoi tessellation were both set at 300. The best K value was inferred based on the run with the highest likelihood (posterior density).

#### 2.3.4 Demographic history estimation

We performed the coalescent-based isolation with migration model using the IMa program (Hey and Nielsen, 2007) to calculate the demographic parameters of the effective population sizes and migration rates for each *A. formosana* population. Simulation studies have shown that the accuracy of the method decreases when analyzing increased numbers of populations (Faubet et al., 2007). To analyze IMa, we grouped the sampling sites into CHA and DAWU and DL. When evaluating the merger of DAWU and DL, we considered the potential bias that could arise due to differences in the sample quantities, as the amount of DL samples significantly differed from CHA and DAWU. Additionally, we also took into account the results of the genetic structure analysis using STRUCTURE and InStruct. For the Isolation-with-Migration model (IMa) (Hey and Nielsen, 2007), the Stepwise Mutation Model (Kimura and Ohta, 1978) was used. The effective population size parameter (*q*) was assigned to all present and ancestral populations. Additionally, migration rate parameters (*m*) between source and target populations were provided for all pairs. The mutation rate of the microsatellite of the directly related species of *A. formosana* has not been calculated. Therefore, the mutation rate of the existing record of plant microsatellites was used as a reference. Specifically, the mutation rate of 6.3×10^-4^ (ranging from 3.0×10^-5^ to 4.0×10^-3^) of the Thuja plicata was selected for related coefficient conversion (O’Connell and Ritland, 2004). MCMC runs were carried out with a burn-in of 2 million iterations, followed by an additional 20 million iterations of the Markov chain.

As an alternative to the IMa model, the linkage disequilibrium method (gametic phase disequilibrium) was done using LDNe v1.31 (Waples and Do, 2008) for calculating all populations’ contemporary effective population size. We excluded rare alleles with frequencies less than three different threshold values (Pcrit of 0.05, 0.02 and 0.01) (Waples, 2006) and obtained 95% confidence intervals for *Ne* by using the jackknife option (Waples and Do, 2008).

Two different methods were used to estimate the migration rate: IMa and non-equilibrium Bayesian MCMC method in BayesAss software (v3.0.4). BayesAss estimates recent migration rates among populations within the past few generations (Wilson and Rannala, 2003). This method accurately detects recent low migration rates within a population by precisely comparing genotype disequilibrium with sampled populations without making any assumptions about Hardy-Weinberg equilibrium. We conducted ten separate runs using different initial random seed numbers to ensure consistent results. The Markov Chain Monte Carlo method was utilized for 10 million iterations, with a 1 million burn-in length. The MCMC phase was sampled every 2000 steps. The delta values for allele frequency, migration rates, and level of inbreeding were kept at 0.15.

## 3 Results

### 3.1 Low genetic variability within and across the three populations

A genomic locus under selection force will show a pattern of away from neutrality, and it is unsuitable for evaluating genetic diversity and population structures. The neutrality of the 15 microsatellites was assessed by calculating the fixation index (*F_ST_*) in all 678 samples (**Supplementary Figure S1**). The result showed that the Am-3mer-16 locus had excessively high genetic variability and differentiation, falling outside the 95% confidence interval (high *F_ST_*). However, it was not statistically significant (*p* > 0.05) (**Supplementary Figure S1**). Therefore, all 15 loci were considered neutrally evolving and applied to subsequent related analysis research. Among the 15 microsatellite markers analyzed, 14 demonstrated Polymorphism Information Content (PIC) values ranging from 0.4 to 0.9, indicating moderate to high polymorphism. Only one microsatellite locus (Am-3mer-71A) showed a low PIC value of 0.2 (**Supplementary Table S2**). These results suggest that the majority of the selected microsatellite loci exhibit substantial polymorphism in *A. formosana* and are suitable for identifying variations both within and between populations.

The genetic diversities among the three populations (CHA, DAWU, and DL) were analyzed by the number of different alleles (*Na*), the number of effective alleles (*Ne*), observed heterozygosity (*H_O_*), and expected heterozygosity (*H_E_*). The analyses were conducted on a total of 678 samples regardless of geographic regions and further within three populations. The average *Na* and *Ne* of the total population (n= 678) were 7.8 and 3.7, respectively (**Table 1**). The highest and lowest *Na* were 16 from Am-2mer-7-9 and 3 from Am-3mer-71A and Am-3mer-124. The highest and lowest *Ne* were 8.0 from Am-2mer-1-96 and 1.3 from Am-3mer-71A. We identified that the observed heterozygosities (*H_O_*) all significantly differed from the expected heterozygosity (*H_E_*) in all loci: 13 out of 15 loci showed significantly lower *H_O_* than *H_E_* except for the Am-3mer-5 and Am-3mer-239 loci. In particular, heterozygosity was absent in 5 microsatellite loci (*H_O_* = 0.0), indicating a high degree of inbreeding and low genetic variability of the species. Similar genetic diversity and polymorphic patterns were observed independently in the three populations (**Table 1**).

**Table 1:** Estimates of genetic diversity by 15 polymorphic microsatellite loci of the Total and the 3 populations (CHA, DAWU and DL) *A. formosana*.

Locus	Total (n=678)				CHA (n=383)				DAWU (n=228)				DL (n=67)			
	*Na*	*Ne*	*H_O_*	*H_E_*	*Na*	*Ne*	*H_O_*	*H_E_*	*Na*	*Ne*	*H_O_*	*H_E_*	*Na*	*Ne*	*H_O_*	*H_E_*
Am-3mer-5	5.0	2.2	0.8	0.5***	5.0	2.3	0.8	0.6***	4.0	2.1	0.9	0.5***	3.0	2.1	1.0	0.5***
Am-3mer-14	8.0	2.5	0.3	0.6***	6.0	2.2	0.2	0.6***	7.0	2.8	0.4	0.6***	5.0	2.5	0.2	0.6***
Am-3mer-16	8.0	4.4	0.4	0.8***	7.0	3.2	0.6	0.7***	6.0	2.9	0.1	0.7***	4.0	1.7	0.0	0.4***
Am-3mer-71A	3.0	1.3	0.0	0.2***	3.0	1.5	0.0	0.3***	2.0	1.1	0.0	0.1***	2.0	1.1	0.0	0.1***
Am-3mer-71B	5.0	2.1	0.0	0.5***	3.0	1.7	0.0	0.4***	4.0	2.5	0.0	0.6***	3.0	2.3	0.1	0.6***
Am-3mer-114	6.0	2.8	0.0	0.7***	6.0	2.5	0.0	0.6***	3.0	2.3	0.0	0.6***	3.0	2.4	0.0	0.6***
Am-3mer-117	8.0	4.4	0.2	0.8***	7.0	4.4	0.3	0.8***	3.0	2.0	0.1	0.5***	3.0	1.8	0.0	0.4***
Am-3mer-118	7.0	4.5	0.1	0.8***	5.0	3.4	0.1	0.7***	6.0	2.3	0.2	0.6***	3.0	2.6	0.0	0.6***
Am-3mer-124	3.0	2.1	0.0	0.5***	3.0	2.3	0.0	0.6***	2.0	1.9	0.0	0.5***	2.0	1.4	0.0	0.3***
Am-3mer-143	4.0	2.0	0.0	0.5***	4.0	2.1	0.0	0.5***	2.0	1.9	0.0	0.5***	2.0	2.0	0.0	0.5***
Am-3mer-197	8.0	3.2	0.0	0.7***	7.0	3.4	0.0	0.7***	7.0	2.6	0.0	0.6***	5.0	2.8	0.0	0.6***
Am-3mer-239	10.0	5.3	0.9	0.8***	9.0	5.0	0.9	0.8***	7.0	5.1	1.0	0.8***	6.0	2.9	1.0	0.7***
Am-2mer-1-60	14.0	4.9	0.4	0.8***	9.0	4.9	0.4	0.8***	11.0	3.8	0.5	0.7***	8.0	3.0	0.4	0.7***
Am-2mer-1-96	12.0	8.0	0.7	0.9***	10.0	5.9	0.8	0.8***	9.0	5.3	0.6	0.8***	10.0	5.3	0.5	0.8***
Am-2mer-7-9	16.0	5.3	0.5	0.8***	14.0	4.9	0.6	0.8***	10.0	4.0	0.3	0.8***	7.0	3.4	0.5	0.7***
Mean	7.8	3.7	0.3	0.7	6.5	3.3	0.3	0.6	5.5	2.8	0.3	0.6	4.4	2.5	0.2	0.5

***Deviation from Hardy-Weinberg equilibrium: *p <*0.001.

Number of different alleles (*Na*), number of effective alleles (*Ne*), observed heterozygosity (*H_O_*) and expected heterozygosity (*H_E_*) are listed.

### 3.2 The genetic differentiation and spatial genetic structure

Genetic differentiation and diversity were analyzed at different levels. First, the analysis treated all collected populations and sample plots as one hierarchical group with different subpopulations (i.e. Cha-1, Cha-2, Cha-3, Cha-4, Cha-5, DAWU and DL). Next, the analyses were performed on the three populations, CHA, DAWU, and DL, as three hierarchical groups. Consequently, we aimed to identify different levels of genetic variations between and within populations of the three geographic regions.

The analyses on the total population (one hierarchical group) showed that the accumulated genetic variations mainly existed within populations (47.9%, *F_IS_* =0.5) and within individuals (40.8%) (**Table 2A**). This indicated the presence of subpopulations within different populations. The second level of analysis (three hierarchical groups) indicated that the majority of genetic variations were observed within subpopulations (87.7%) (**Table 2B**).

**Table 2:** Analysis of molecular variance (AMOVA) for 678 individuals based on 15 microsatellite loci in *A. formosana*.

Source of variation	Sum of squares	Variance components	% of Variation	Fixation indexes
(A) One hierarchical group				
Among populations	451.3	0.6	11.3	*F_ST_* =0.1*
Among individuals and within populations	4632.3	2.5	47.9	*F_IS_* =0.5*
Within individuals	1372.5	2.1	40.8	*F_IT_* =0.6*
(B) Three hierarchical groups				
Among groups	397.4	0.4	7.8	*F_ST_* =0.1*
Among populations and within groups	53.9	0.2	4.6	*F_SC_* =0.1*
Within populations	6004.8	4.6	87.7	*F_CT_* =0.1

(A) The analysis treated total samples (n=678) as a single hierarchical group. (B) The analysis of the samples from three geographic locations as independent hierarchical groups. Results include the sum of squares, variance components, percentage of variance, and fixation indexes. * *p* < 0.05.

The *F*-statistics indicated moderate genetic differentiation among populations and among groups (*F_ST_* =0.1 and 0.1, **Table 2**). The results showed that both *F_IS_* and *F_IT_*, indicators to detect the degree of inbreeding in the population, were significantly greater than 0 (*F_IS_* =0.5, *F_IT_* =0.6, *p* < 0.05). It indicated the existence of a significant degree of inbreeding within populations. The genetic differentiation among populations within groups (*F_SC_*) was low but significant (*p* < 0.05), indicating potential subpopulation structure within different geographic regions (groups).

Taken together, the results consistently pointed out cryptic population structures of the three populations and were in line with the low genetic variability as indicated by low or no observed heterozygosity.

### 3.3 Three independent analyses reveal cryptic genetic structures

As *F*-statistic indicated potential cryptic genetic structures of the species, we further applied three different analyses, STRUCTURE, InStruct and GENELAND, to identify population structures in different geographic regions. We described the results of the three analyses in the following subparagraphs. For the cluster code, we assigned T, C, D, and L representing Total sample (T), CHA population (C), DAWU population (D), and DL population (L), followed by S, I, and G representing STRUCTURE (S), InStruct (I), and GENELAND (G) analysis results. For example, TS-2-1 represents the first cluster (1) of the two (K=2) clusters of the total sample (T) using STRUCTURE (S) clustering analysis.

#### 3.3.1 STRUCTURE analysis

**Figure 2:**
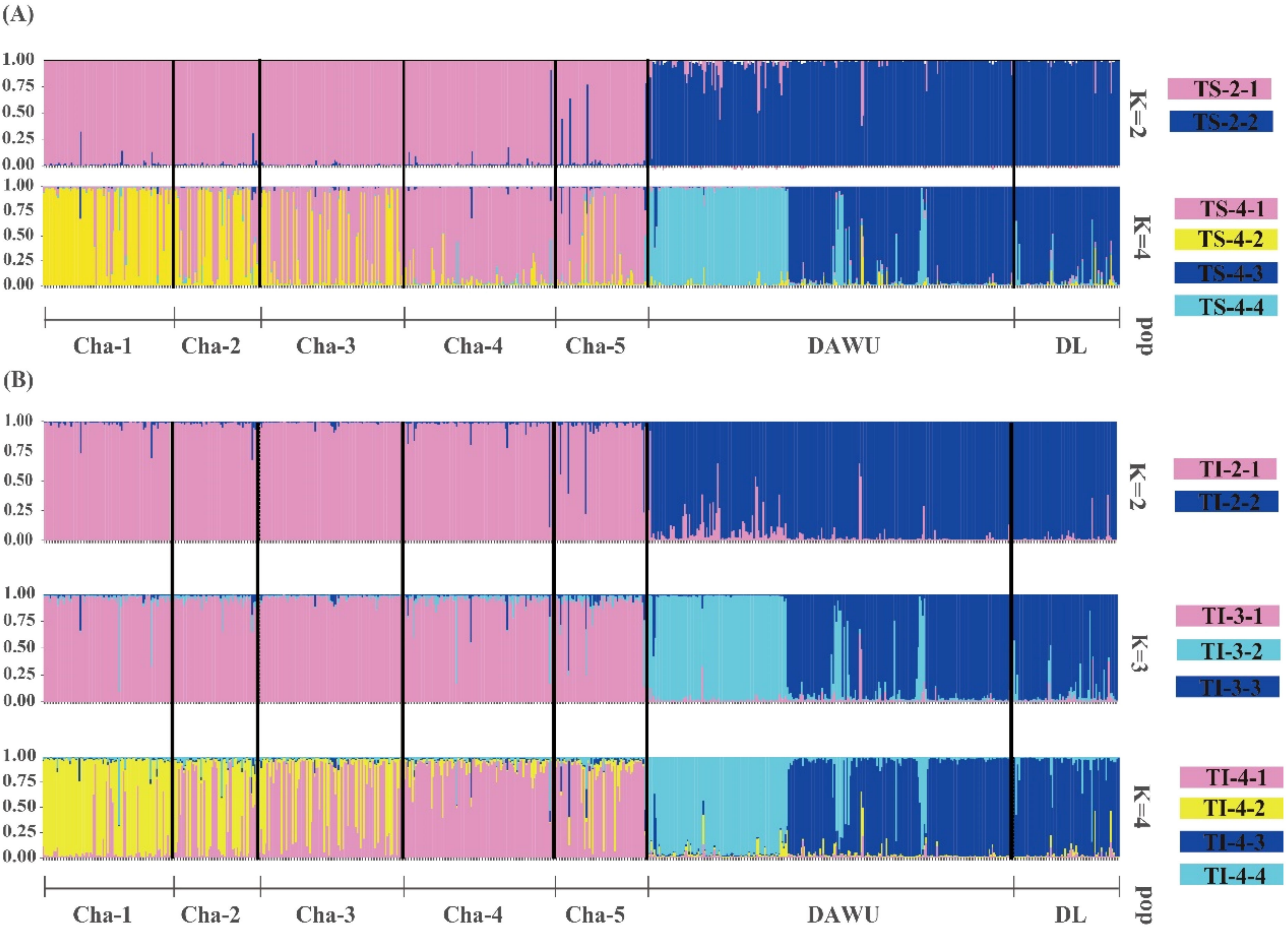
Bar plots showing the genetic structures of all sampling sites (n=678 from CHA, DAWU and DL) of *A*. *formosana* based on STRUCTURE **(A)** and InStruct **(B)** analyses. A vertical color line represented each individual, and the same color indicated that the individual belonged to the same cluster.

**Figure 3:**
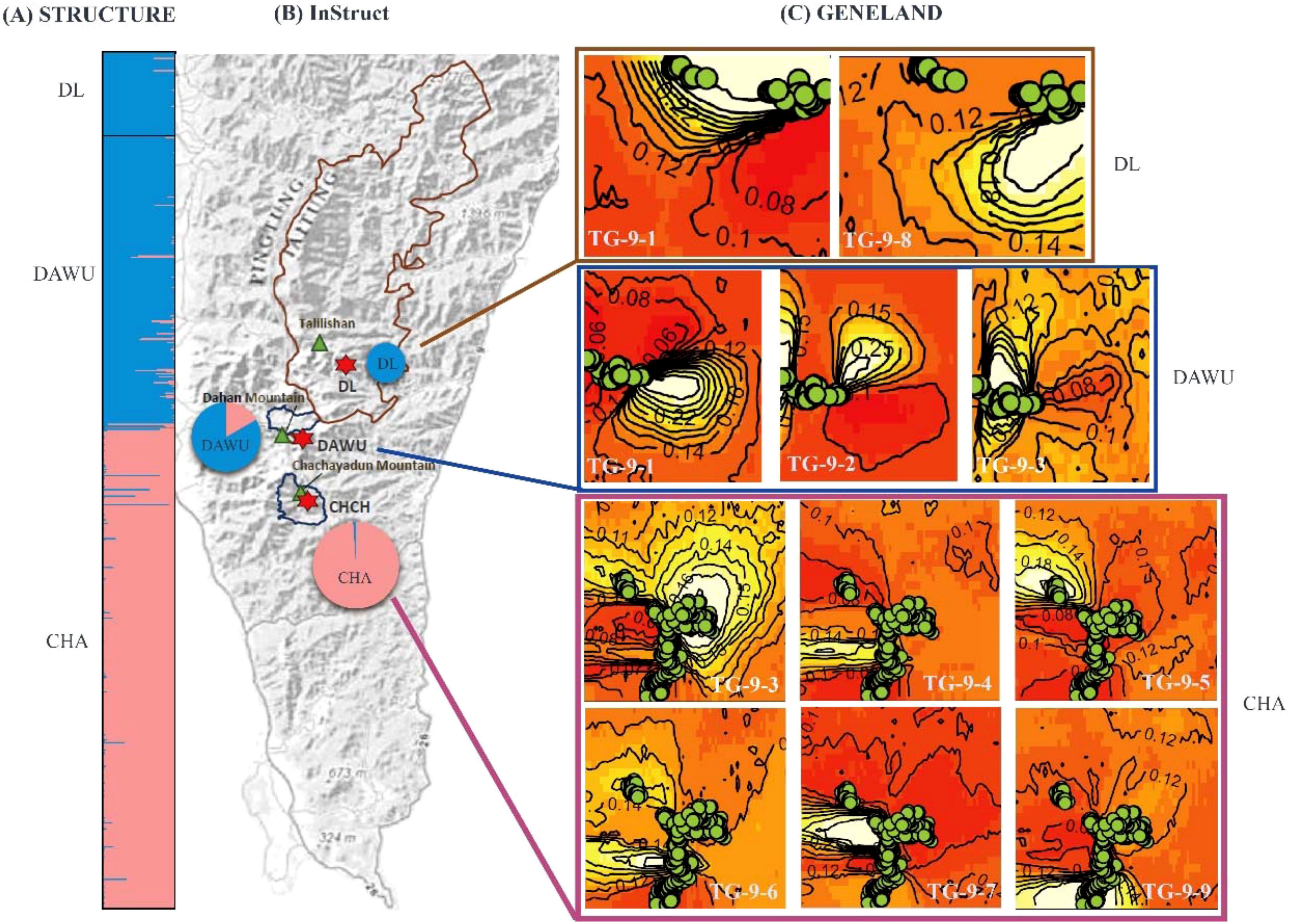
Bayesian clustering analysis of all sampling sites (CHA, DAWU, and DL populations) of *A*. *formosana* using three analyses. **(A)** STRUCTURE analysis: the result of 2 clusters (K=2) was shown. **(B)** InStruct analysis: the pie charts show the frequencies of the 2 clusters in each of the three populations (K=2), with the size of each pie chart representing the relative sample population size. **(C)** GENELAND analysis: the green circle represents the sampling sites, and 9 clusters were identified. The gradient from light (white) to dark (red) indicates a high to low probability of membership in a particular subpopulation. The labels TG-9-1 through TG-9-9 represent the genotype codes.

The clustering analysis performed using STRUCTURE determined the optimal number of clusters (K) based on the magnitude of ΔK. Among all sampling sites, the best K was found to be 2 clusters (cluster code TS-2), followed by 4 clusters (cluster code TS-4) (**Figure 2**; **Supplementary Table S3**). The results of both clustering showed that the DAWU and DL populations were distinguishable from the CHA population, suggesting the presence of unique genotypes in different populations with minimal intermixing, leading to distinct genetic groups (**Figures 2A**, **3A**). With 4 clusters, distinct genotypes were detected within the three populations, indicating the presence of hidden subpopulation structures, especially in the CHA and DAWU populations (**Figure 2A**). Thus, the CHA, DAWU, and DL populations were further analyzed independently to uncover cryptic genetic groups in detail.

The independent clustering results of STRUCTURE showed that the highest Δ K value was observed for two clusters (cluster code: CS-2) across all three populations. Further subdivision resulted in three clusters each in the CHA (cluster code: CS-3) and DAWU (cluster code: DS-3) populations and six clusters in the DL population (cluster code: LS-6) (**Supplementary Table S3**; **Supplementary Figures S2**, **S3**). Notably, the CHA population exhibited potential for further subdivision into more clusters, with K values of 4, 5, 7, and 8 (**Supplementary Figure S2A**; **Supplementary Table S3**). Of particular significance, the K=8 clustering result was consistent with the subsequent GENELAND clustering results, as will be described in the later paragraph (**Supplementary Figure S4**).

#### 3.3.2 InStruct analysis

The InStruct analysis, aligned with the STRUCTURE results, indicated the optimal number of clusters across all three populations to be 2 clusters (cluster code: TI-2), followed by 3 (cluster code: TI-3) and 4 (cluster code: TI-4) clusters (**Figures 2B**, **3B**; **Supplementary Table S4**).

The independent clustering results of each population also showed 2 clusters (cluster code: CS-2, DS-2 and LS-2) as the optimal clustering with the highest ΔK value (**Supplementary Table S4**). Nevertheless, when considering both likelihood and DIC value results, the optimal clustering was found to be K=18 (cluster code: CI-18), 13 (cluster code: DI-13), and 8 (cluster code: LI-8) in the CHA, DAWU, and CL populations, respectively (**Supplementary Table S4**).

#### 3.3.3 GENELAND analysis

The GENELAND analysis indicated that the optimal number of clusters with the highest credibility across all three populations was 9 (over 40% probability, cluster code: cluster code: TG-9) (**Figure 3C**). Among the 9 clusters, TG-9-1 was shared by the DL and DAWU populations, while the CHA and DAWU populations shared TG-9-3 and several unique genotype compositions were found within the CHA population (TG-9-4, TG-9-5, TG-9-6, TG-9-7, TG-9-9) along with TG-9-2 in DAWU and TG-9-8 in DL. Overall, GENELAND provided more insight into identifying cryptic structures compared to STRUCTURE and InStruct.

The independent clustering results of the three populations showed that each population could be subdivision into 8 (CHA, cluster code: CG-8), 15 (DAWU, cluster code: DG-15), and 6 (DL, cluster code: LG-6) clusters (**Supplementary Figure S5**). In particular, the clustering results of the DL population are consistent with the independent clustering results obtained using STRUCTURE.

### 3.4 Significant reduction and minimal gene flow between subpopulations

Assessing effective population size is crucial in determining populations’ ability to maintain genetic diversity and developing conservation strategies. To understand the degree of population contraction of the species, we used two different analyses, IMa and LDNe, to estimate the effective population size. Additionally, gene flow between populations was estimated using IMa and BAYESASS analysis.

For IMa analysis, considering the potential bias that could arise due to differences in sample size and factoring in results from genetic structure analysis using STRUCTURE and InStruct, DAWA and DL populations were grouped into one DAWU/DL population for the analysis. The results of the IMa analysis showed that the effective population size of the ancestor (N_A_) was 16,145.2 (95% CI: 12804.8-17258.7), which was significantly greater than current populations of CHA (N_1_ = 27.8, 95% CI: 9.3-250.5) and the combined DAWU/DL population (N_2_ = 9.8, 95% CI: 4.2-1131.9) (**Supplementary Table S5**; **Supplementary Figure S6**).

Effective population sizes estimated by LDNe excluded rare alleles with minimum allele frequencies less than 0.05, 0.02 and 0.01, respectively (**Supplementary Table S6**). Across total populations, the effective population size (*Ne*) are 39.7 (95% CI: 30.8-50.3), 49.1 (95% CI: 39.8-59.9), and 54.7 (95% CI: 44.3-66.8) under conditions of cut-off 0.05, 0.02 and 0.01, respectively. CHA population had an *Ne* of 94.4 (95% CI: 74.4-121.0), 102.1 (95% CI: 78.7-134.2), and 105.1 (95% CI: 81.8-136.9). DAWU population had a *Ne* of 23.4 (95% CI: 16.3-32.7), 28.7 (95% CI: 20.4-39.6), and 36.5 (95% CI: 26.8-49.6). DL population had a *Ne* of 33.0 (95% CI: 22.7-50.8), 37.9 (95% CI: 25.5-60.8), and 45.1 (95% CI: 29.4-77.1).

Historical patterns of gene flow revealed by IMa showed generally low levels of gene flow (**Supplementary Table S5**; **Supplementary Figure S6**). The gene flow from the CHA (M_1_) to the DAWU/DL (M_2_) (M_1→2_ = 0.5^-03^, 95% CI: 0.2^-03^~0.1) was slightly larger than in the opposite direction (M_2→1_ = 0.1^-03^, 95% CI: 0.6^-03^-0.1) (**Supplementary Table S5**; **Supplementary Figure S6**). Such asymmetric gene flow was also detected in contemporary migration events. Using BAYESASS v3.0.391 to infer the contemporary migration rate, migration rates among different populations of *A. formosana* were generally low (**Supplementary Table S7**). The rate of gene flow from the CHA population to the DAWU/DL populations were 0.1^-02^ (95% CI: 4.9^-05^-0.5^-02^) and 0.3^-02^ (95% CI: 2.1^-05^-0.1^-01^), while the rates in the opposite direction were 0.5^-03^ (95% CI: 6.3^-06^-0.2^-02^) and 0.6^-03^ (95% CI: 4.3^-06^-0.3^-02^) respectively. Gene flow from DL to DAWU (0.2, 95% CI: 1.7^-01^-2.2^-01^) was higher than in the opposite direction (0.3^-02^, 95% CI: 2.6^-05^-0.1^-01^) (**Supplementary Table S7**).

### 3.5 Delineation of management units and selection for *ex situ* conservation of trees within the monitoring regions

Genetic conservation units can be identified using various approaches considering historical (Evolutionary Significant Units, ESUs) or current population structures (Management Units, MUs). Given that most speciation events for *Amentotaxus* species were relatively recent (around 2.5 million years ago) (Ge et al., 2014), we proposed using the MUs concept to define genetic conservation units in this study. This study aims to combine ecological survey data on Diameter at Breast Height (DBH) and forest health indexes with genetic structure information to define relevant MUs for fine-scale genetic conservation.

We recommended that the three habitats of *A. formosana* should be managed independently for the most comprehensive conservation strategy, and MUs should be defined based on the results of GENELAND analysis from the three populations independently for the following reasons: (1) cryptic population structures were identified with confidence in both the CHA and DAWU populations through all three analysis methods (**Figures 2**, **3**); (2) STRUCTURE and InStruct do not identify unique subpopulations in DL from DAWU and CHA populations, while GENELAND analysis confirms the unique subpopulations in DL (TG-9-8) (**Figure 3**); (3) more detailed clustering of cryptic structures were identified when the analyses applied independently to the three populations (**Supplementary Figures S2**, **S3**, **S5**).

We suggested using K=8, 15, and 6 for CHA, DAWU, and DL populations, respectively, based on the independent clustering results of the GENELAND analysis (**Supplementary Figure S5**) for the most comprehensive delineation of management units for the species in Taiwan. Therefore, we designated the CG-8-1 to CG-8-8, DG-15-1 to DG-15-15, and LG-6-1 to LG-6-6 in CHA, DAWU, and DL population, respectively, as distinct MUs (**Supplementary Figure S5**; **Supplementary Table S8**). This will benefit future genetic conservation management strategies and ensure the comprehensive coverage of unique subpopulation units of the species.

At least three middle-aged trees from different genetic conservation units will be selected for *ex-situ* conservation through cutting propagation to preserve maximum genetic diversity. According to the literature, *A. formosana* trees are typically around 60-80 years old, and trees with a DBH of 5 cm or more are considered middle-aged trees (Chan, 2005). The criteria for identifying middle-aged trees is based on a survey by Chan (2005), which indicated that *A. formosana* trees are typically around 60-80 years old, and every 20 years, 1-2 seedlings may grow to a diameter of over 5 cm (Chan, 2005). As a result, trees with a DBH of 5-10 cm are considered middle-aged trees in this study and are our primary selection focus. We measured the DBH of trees at all sampling sites and found that 79.7% of the samples belonged to middle-aged trees (**Supplementary Figure S7A**).

The health of *A. formosana* is another key factor in determining which individuals should be selected for genetic conservation. The health standards information is based on a report by Ho et al. (2012c), which defines the health grading standards for *A. formosana* as follows: “1” for severely unhealthy or dead, “2” for unhealthy, “3” for beginning to deteriorate, “4” for healthy, and “5” for very healthy (Ho et al., 2012c). Therefore, individuals with health levels 4 and 5 are given priority for conservation selection in this study. This classification was applied specifically to the CHA population, revealing that 55.2% of the individuals in this study are at healthy levels 4 and 5.

**Figure 4:**
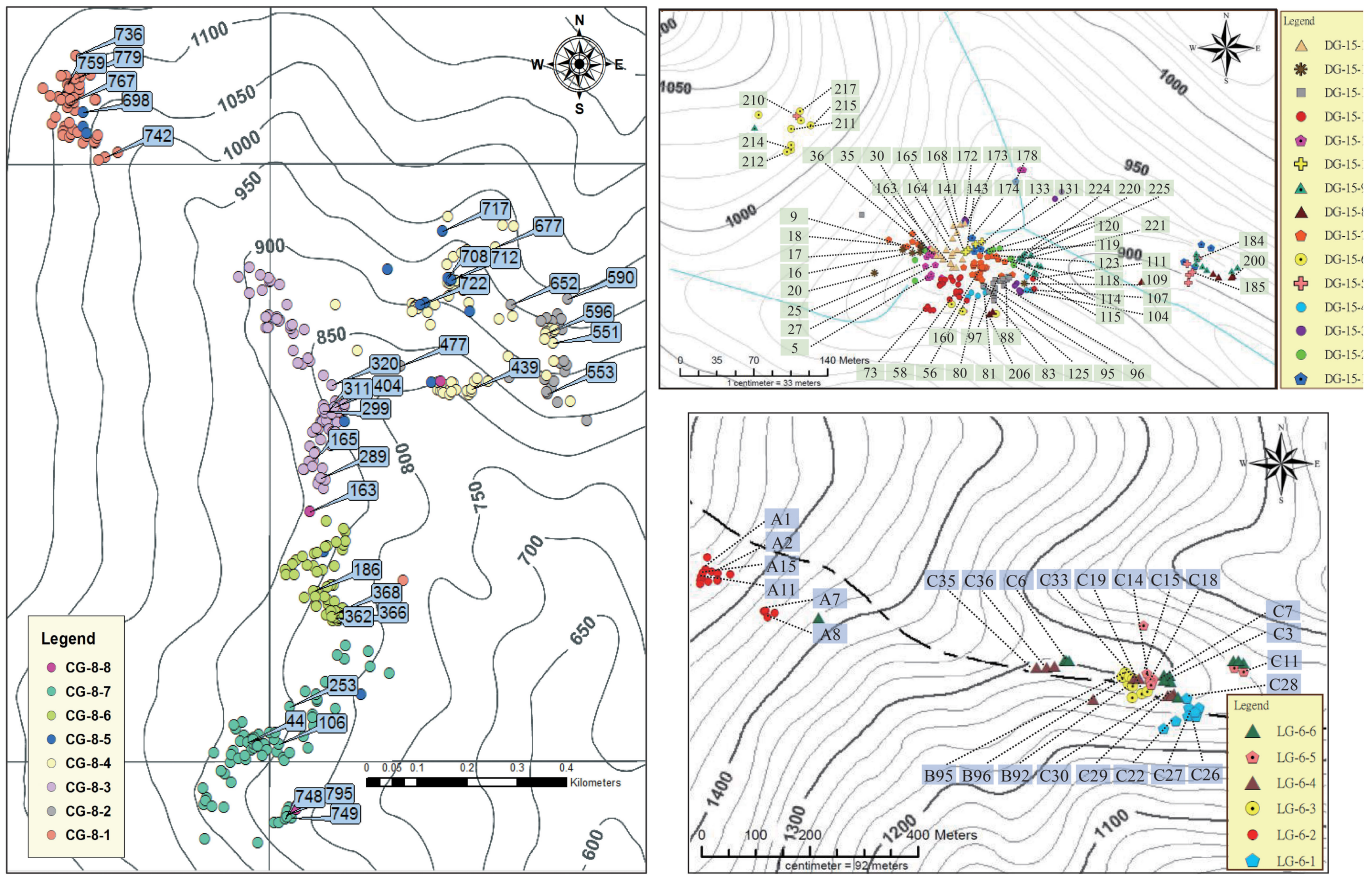
The distribution and selection of management units and *ex-situ* genetic conservation trees of *A. formosana* in CHA, DAWU and DL populations. The sample numbers of trees are provided within the text.

Considering the above-defined MUs, DBH and health grading standards, specific criteria were established for selecting trees for *ex-situ* conservation. As a result, 35, 59, and 26 individuals from the CHA, DAWU, and DL, respectively, were selected for future *ex-situ* genetic conservation management (**Figure 4**; **Supplementary Table S8**).

## 4 Discussion

### 4.1 Level of genetic diversity and the problem of inbreeding

Genetic diversity is a crucial factor in determining management units. Fifteen polymorphic microsatellite markers previously developed for *A. formosana* (Ho et al., 2012a) and were used to assess genetic variation in the Chachayalaishan mountain region, showing low diversity in the local population (Ho et al., 2012b). These markers have since been used to study the species complex’s phylogeny and *A. argotaenia*’s genetic differentiation (Ge et al., 2014; Nguyen et al., 2022). Our analysis of *A. formosana* populations from various regions using these markers revealed notable deviations from Hardy-Weinberg equilibrium due to a heterozygote deficiency. While the average number of different alleles (*Na*) was higher than the *A. yunnanensis* (Miao et al., 2008) and *Taxus wallichian* (Yang et al., 2009) but lower than other gymnosperm species such as *Pinus pinaster*, *P. sylvestris*,*Taxus baccata*, and *Keteleeria davidiana* var. *formosana* (Mariette et al., 2001; Scalfi et al., 2009; Dubreuil et al., 2010; Ho et al., 2014). Interestingly, despite similar population sizes between CHA and DAWU regions (748 and 762 individuals, respectively) as shown by two independent surveys (Yang et al., 2008; Pingtung Forestry Management Office, 2009), our study revealed higher genetic diversity in the CHA population compared to the DAWU population. This discrepancy between population size and genetic diversity highlights the critical importance of integrating genetic information in conservation efforts for *A. formosana*.

The observed reduction in heterozygosity in *A. formosana* populations is likely caused by inbreeding, leading to genetic drift and decreased population fitness. High *F_IS_* and *F_IT_* values also indicate significant inbreeding in the *A. formosana* population. Three main factors contribute to this inbreeding: (1) reproductive strategy, (2) limited pollen and seed dispersal, and (3) habitat fragmentation. Firstly, *A. formosana* primarily reproduces asexually. Its weak sexual reproduction results in few seedlings in the field (Yang et al., 2008), increasing the likelihood of mating between close relatives. Secondly, the species’ height rarely exceeds the mid-canopy layer of 15 m, and it’s primarily shade-tolerant at the lowest canopy layer (Yang et al., 2008). While this reduces the impact of strong monsoons, it may limit pollen dispersal (Hamrick and Godt, 1996; Hamrick and Nason, 2000). The year-round humid habitat may also hinder pollen release (Yang et al., 2008). Moreover, its large, heavy seeds are not readily dispersed to distant places, leading to clumped distribution near the parent tree and potential inbreeding (Yeh et al., 1992; Yang et al., 2008). The low-efficiency pollen and seed dispersal leading to inbreeding in the population also occurs in other *Taxaceae* species and some temperate forest tree species (Chaisurisri and El-Kassaby, 1994; González-Martínez et al., 2010; Chybicki et al., 2011). Lastly, habitat fragmentation impedes pollen and seed dispersal, resulting in high differentiation and reduced genetic diversity. The negative impact of habitat fragmentation on genetic diversity has been observed in many woody plants (Jump and Peñuelas, 2006; Knapp et al., 2001; Vranckx et al., 2012). Fragmented habitats lead to geographic isolation, reduced migration, decreased gene exchange, and smaller effective population sizes (Couvet, 2002; Aguilar et al., 2008). These factors increase the risk of genetic drift (Ellstrand and Elam, 1993) and inbreeding (Keller and Waller, 2002), ultimately leading to the Wahlund effect (Allendorf and Luikart, 2007).

### 4.2 Dynamics of effective population size and degree of gene flow

Understanding the effective population size is crucial for conservation genetics, as it differs from the actual surveyed population size. The impact on genetic diversity when a population is threatened largely depends on its effective size rather than its census size (Frankham et al., 2010; Bruford et al., 2017). Our analysis using IMa revealed that the CHA population maintains a significantly higher effective population size than the DAWU and DL populations. However, when compared to ancestral populations, all current *A. formosana* populations show substantial reductions, indicating a concerning trend of population decline. This finding is further supported by LDNe analysis, which demonstrates generally low and variable effective population sizes across the studied populations. It’s worth noting that while field surveys have reported thousands of individual trees (Yang et al., 2008; Pingtung Forestry Management Office, 2009), genetic assessments paint a different picture, highlighting the importance of genetic data in conservation planning.

The causes of this population decline are likely multifaceted, but habitat fragmentation and environmental changes appear to be primary factors. These pressures have reduced population sizes and limited gene flow between populations. Both historical and contemporary gene flow assessments show extremely low levels of genetic exchange among populations. This isolation, combined with small effective population sizes, puts *A. formosana* at high risk of inbreeding depression and local extinction, even without direct human interference. Interestingly, AMOVA results indicate that most genetic variation in *A. formosana* exists within populations, with relatively low genetic differentiation between populations. The shallow divergence time between populations suggests they have only recently become isolated. However, given the limited gene flow and reduced effective population sizes, we predict inter-population differentiation will increase over time, potentially leading to more distinct subpopulations.

This demographic history underscores the urgent need for conservation interventions. Management strategies should focus on maintaining and increasing effective population sizes, as well as facilitating gene flow between isolated populations to mitigate the effects of genetic drift and inbreeding.

### 4.3 Patterns of genetic structure

Gymnosperm plants, particularly those with long generations and easy cross-pollination, often exhibit significant fine-scale genetic structure within short distances (Pandey and Rajora, 2012). This genetic structure is especially vulnerable to human interference and population fragmentation, as observed in species like *Thuja occidentalis* (Pandey and Rajora, 2012), *Pinus elliottii* var. *densa* (Williams et al., 2007), and *Pinus pinaster* (De-Lucas et al., 2009). These species typically have mixed-mating systems with high selfing levels and limited seed dispersal, leading to larger neighborhood sizes in core populations than peripheral ones, resulting in a block-like presentation of genetic partitioning (Pandey and Rajora, 2012). Therefore, understanding the genetic structure to guide the design of small-scale conservation units of endangered species is becoming increasingly urgent.

Our study on *A. formosana* revealed significant genetic differentiation among the CHA, DAWU, and DL populations despite similarities in their ecological environments (Jiang et al., 1995; Yang et al., 2008). Interestingly, Wu and Luo (1992) found that morphological variations in *A. formosana* were more influenced by maternal inheritance than micro-habitat conditions (Wu and Luo, 1992). This suggests that the genetic structure of *A. formosana* may not be directly related to current environmental conditions but somewhat shaped by historical factors, particularly population bottlenecks during and after Pleistocene glaciations, Holocene warming-induced habitat shifts, and subsequent restricted dispersal and gene flow (Ge et al., 2014).

The genus *Amentotaxus* was once widely distributed in the northern hemisphere (Farjon, 2001; Follieri, 2010) but experienced a decline and became scattered during the Pleistocene and Holocene epochs (Ferguson et al., 1978; Royer et al., 2003). Taiwan, which emerged above sea level in the Pleistocene and reached peak warmth about 6,000 years ago (He et al., 2004), likely provided refuge for *A. formosana* during the Ice Age (Ge et al., 2014). We hypothesize that Holocene warming triggered population declines and forced retreats to high-altitude areas. This resulted in a bottleneck effect, limiting the current population size and leading to sparse distribution along the Central Mountain Range. These factors, coupled with reproductive strategy issues and inbreeding, resulted in limited gene flow. Consequently, the current population structure exhibits significant differentiation across habitats and contains multiple subpopulations within populations.

Our GENELAND analysis revealed 6 to 15 distinct genetic groups within the three *A. formosana* populations, with genetic structures partially matching sampling plot designations. This indicates that the genetic structure within the population is not wholly randomly distributed. This discovery of distinct genetic structures within the population is a novel finding for *Amentotaxus* species. While Nguyen et al. (2022) found genetic differentiation among geographically segregated populations of *A. argotaenia*, they did not detect within-population genetic structure (Nguyen et al., 2022). Although population differentiation may be prevalent in *Amentotaxus* species, it has not been extensively studied previously. The multiple genetic structure patterns of *A. formosana* proposed in this study can serve as valuable references for future genetic structure research on other *Amentotaxus* species.

### 4.4 Refining management units to enhance genetic conservation strategies

Low genetic diversity within a species is a major factor that increases extinction risk, particularly with the ongoing environmental changes (Fricke et al., 2022). Therefore, it is essential to safeguard genetic variations and distinctiveness to conserve endangered species. A vital step towards this goal is to divide the existing populations into different conservation units as part of conservation practices (Allendorf and Luikart, 2007). To effectively conserve endangered species like *A. formosana*, it is essential to safeguard genetic variations and distinctiveness. This study combines genetic structure data with ecological surveys to assess the health status of *A. formosana* in the wild and identify genetic conservation units that cover all ecological situations where the species is found.

Previous research on the *A. argotaenia* species complex, including *A. formosana*, has utilized various genetic markers to clarify genetic divergence and diversity (Wang et al., 1996; Ge et al., 2005; Ho et al., 2012b; Ge et al., 2014; Nguyen et al., 2022). Ge et al. (2014) analyzed the genetic divergence of the species complex using chloroplast DNA, mitochondrial DNA, and multiple microsatellite markers (Ge et al., 2014). While results for mainland China species were inconsistent across different markers, *A. formosana* consistently emerged as monophyletic in phylogenetic analyses. Studies also revealed low genetic diversity and divergence within and between populations of the four species, attributed to demographic bottlenecks during and/or after the Pleistocene glaciations (Wang et al., 1996; Ge et al., 2005, 2014). The consistent monophyletic status and genetic uniqueness of *A. formosana* support its classification as an independent Evolutionary Significant Unit (ESU).

Our study partially supports considering different *A. formosana* habitats as genetically distinct units. The management units for *A. formosana* are defined based on GENELAND clustering analysis results, encompassing all unique genotypes. Olsen et al. (2014) found that GENELAND can detect additional genetic units that STRUCTURE cannot, largely aligning with distance-based and non-genetic methods (Olsen et al., 2014). Based on GENELAND analysis results, we identified 8, 15, and 6 management units (MUs) in the CHA, DAWU, and DL populations. Individual MUs contain distinct genetic profiles across diverse mountainous terrain, necessitating the preservation of both genetic and ecological diversity. Given this complexity, implementing uniform management protocols across an entire MU would be suboptimal. Instead, developing specialized management strategies tailored to address the specific requirements within each MU represents a more effective approach.

### 4.5 Conservation strategies and management recommendations

*Amentotaxus formosana* inhabits mixed needle-broadleaf misty forests in Taiwan, requiring specific environmental conditions. However, these habitats are vulnerable. Field surveys reveal that its conservation areas face environmental instability. Frequent extreme rainfall and earthquakes cause landslides, making conservation areas fragile. Consequently, the mature population is experiencing a steady decline. Given these challenge, comprehensive conservation approach combining both *in-situ* and *ex-situ* strategies is essential.

For *in-situ* conservation, maintaining the environmental conditions of wild habitats is crucial, particularly focusing on protecting different genetic units within their natural environments. This involves implementing protective measures in each management unit to preserve the species’ natural genetic diversity and ecological adaptations. Special attention must be given to facilitating natural seed and seedling development within each management unit to reinforce gene flow and maintain high genetic diversity while reducing inbreeding depression risks.

*Ex-situ* conservation has become increasingly important, especially given the challenging access to these geologically sensitive and fragile landslide areas. Our study has initiated a significant *ex-situ* conservation effort by selecting 120 individual trees across the CHA (35), DAWU (59), and DL (26) populations. While seed collection proves challenging, field observations have shown that *A. formosana* can propagate through sprout growth, making stem-cutting propagation a viable alternative. These selected trees will be used for cutting experiments and cultivation to establish genetic conservation nurseries and seed stands. This approach not only helps preserve genetic diversity but also provides opportunities for future research on adaptive differentiation between MUs under changing environmental conditions, which will further inform and refine conservation strategies.

## 5 Conclusion

In conclusion, this study provides critical insights into genetic conservation and population dynamics of the endangered species *A. formosana* in Taiwan. Our findings reveal declining effective population sizes, limited gene flow, and fine-scale genetic structures, highlighting the challenges of inbreeding and habitat fragmentation. We identified distinct management units within populations, emphasizing the need for tailored conservation approaches to preserve genetic uniqueness and maintain diversity within each population. By integrating genetic data with ecological surveys and selecting individuals for *ex-situ* conservation, we establish a foundation for informed decision-making. Our comprehensive approach aims to ensure the long-term survival and adaptability of *A. formosana* through proactive measures focused on preserving genetic diversity.

## Data Availability

The original contributions presented in the study are included in the article/**Supplementary Material** as **Raw Data Sheet S1**. Further inquiries can be directed to the corresponding author.

## Supplementary material

Supplementary Table 1 Sampling locations, year, coordinates and sample number in *Amentotaxus formosana* samples.

Supplementary Table 2 Repeat motifs, primer sequence, allelic size, optimized annealing temperature (*Ta*) and PIC at 15 polymorphic microsatellite loci from *A. formosana*.

Supplementary Table 3 Mean LnP(K) and ΔK for clusters using Bayesian assignment test in STRUCTURE to infer the number of clusters K in *A. formosana*.

Supplementary Table 4 Mean LnP(K), ΔK and DIC (deviance information criterion) for clusters using Bayesian assignment test in InStruct to infer the number of clusters K in *A. formosana*.

Supplementary Table 5 Estimates the six IMa model parameters in *A. formosana*, The N, M and T are effective population size (*q*), migration rate (*m*) and divergence time (*t*) scaled by the mutation rate. The N_1_ is the effective population size in the CHA population, N_2_ is the effective population size in DAWU and DL population, N_A_ is the effective population size ancestral, M_1→2_ is the migration rate from CHA to DAWU and DL, M_2→1_ is the migration rate from DAWU and DL to CHA and the T is divergence time. The mutation rate was referred to *Thuja plicata* Donn mutation rate for microsatellite was 6.3×10^-4^ (95% confidence interval of 3.0×10^-5^ - 4.0×10^-3^) mutations per locus per generation (O’Connell and Ritland, 2004).

Supplementary Table 6 Results of estimates of the effective population size (*Ne*) via LDNe analysis in *A. formosana*, independent comparisons, overall *r^2^*, expected *r^2^* and percentage of 95% confidence intervals are included in the analysis.

Supplementary Table 7 Estimates of recent migration rates (including 95% confidence intervals) between 3 populations (CHA, DAWU and DL) of *A. formosana* derived from the program BAYESASS.

Supplementary Table 8 The detailed dissection of the STRUCTURE and GENELAND individual genetic structure analysis results for CHA, DAWU, and DL populations of *A. formosana*. Each component of the clusters is examined layer by layer. The percentage representation of samples under each stratified clustering is also presented, giving in-depth insights into the genetic structure. Additionally, the selection of *ex-situ* conservation trees for each Management Unit is displayed, which includes information on the Diameter at Breast Height (DBH) and health grading standards (H).

Supplementary Figure 1 Plot of *F_ST_* versus heterozygosity (*He*) to test the natural selection effect in 15 microsatellite loci using LOSITAN. There is a grey area for neutrality, and the outliers are identified as candidates for directional selection or balancing selection.

Supplementary Figure 2 The results of Bayesian clustering analysis of *A. formosana* in the CHA population using STRUCTURE **(A)**, InStruct **(B)**, and GENELAND **(C)**. The best number of clusters for the STRUCTURE analysis result is K=2 (followed by K=3, 4, 5, 7, 8), the best number of clusters for the InStruc analysis result is K=18, and the best number of clusters for the GENELAND analysis result is K=8. Each color represents a different cluster, and each bar line represents a different individual.

Supplementary Figure 3 The results of Bayesian clustering analysis of *A. formosana* in the DAWU and DL population using STRUCTURE, InStruct. Each color represents a different cluster, and each bar line represents a different individual. The code represented by different color blocks is shown.

Supplementary Figure 4 Histogram from GENELAND analysis showing the average density to inferred K in total populations and three populations of CHA, DAWU and DL separate analysis results in *A. formosana*.

Supplementary Figure 5 Bayesian cluster analysis output maps from GENELAND using microsatellite data for the CHA **(A)**, DAWU **(B)** and DL **(C)** populations of *A. formosana*. Maps of posterior probabilities belong to K=8, 15 and 6 for the three populations, respectively. The blue circle expressed the sampling sites. The light (white) to dark (red) color indicates the high to low probability of membership belonging to a particular subpopulation. Each plaque represents different genotypes within populations.

Supplementary Figure 6 The posterior distributions for **(A)** two current effective population sizes (N_1_ and N_2_), **(B)** ancestral effective population size (N_A_), **(C)** migration rate (M_1→2_ and M_2→1_) and **(D)** divergence time (T) between CHA population (population 1) and DAWU and DL population (population 2) in *A. formosana*. (scaled by the mutation rate, μ=6.3×10^-4^ per generation).

Supplementary Figure 7 Bar chart representation of the Diameter at Breast Height (DBH) **(A)** and Health Grading Standards **(B)** data for *A. formosana* samples collected from different populations in this study.

Supplementary Raw data sheet S1 Raw data of 678 samples from Amentotaxus formosana populations in Taiwan based on amplicon sizes using 15 SSR loci.
