# Supplementary material for: Assessment of genetic conservation units of an endangered glacial relict insular species, Amentotaxus formosana, based on fine-scale genetic structures of multiple fragmented mountainous populations in Taiwan
Source: Front Plant Sci. 2025 Jan 16;15:1512914. doi: 10.3389/fpls.2024.1512914 (PMC11779735; doi:10.3389/fpls.2024.1512914)
Supplement: Supplementary Table 1 — Sampling locations, year, coordinates and sample number in Amentotaxus formosana samples. [file DataSheet2.docx]

Supplementary Material

Supplementary Table S1. Sampling locations, year, coordinates and sample number in *Amentotaxus formosana* samples.

| Sample location | Year | Longitude(E) | Latitude(N) | Sample code | No. of samples  collected |
| --- | --- | --- | --- | --- | --- |
| Chachayalaishan Major Wildlife Habitat, Shih-zih Township, Pingtung County, Taiwan | 2009 | 120°45'58"E | 22°20'13"N | CHA | 383 |
| Dawu Taiwan *Amentotaxus* Nature Reserve, Daren Township, Taitung County, Taiwan | 2011 | 120°45'40"E | 22°23'55"N | DAWU | 228 |
| Talilishan, Laiyi Township, Pingtung County, Taiwan | 2013 | 120°48'57''E | 22°28'55''N | DL | 67 |

Supplementary Table S2. Repeat motifs, primer sequence, allelic size, optimized annealing temperature (*Ta*) and PIC at 15 polymorphic microsatellite loci from *A. formosana*.

| Locus | Repeat motif |  | | Primer sequence (5' - 3' ) | Fragment size (bp) | *Ta* ( ℃) | GenBank accession no. | PIC |
| --- | --- | --- | --- | --- | --- | --- | --- | --- |
| Am-3mer-5 | (CAA)_8_ | F: | TAGAGATCAGTTGCAGGGA | | 188 | 60 | HE582008 | 0.4 |
|  |  | R: | GGAGTCTACTTACCCTAGGAG | |  |  |  |  |
| Am-3mer-14 | (AT)_6_(CA)_14_(TA)_5_ | F: | TGATCGAACTAGCAGTGGT | | 238 | 53 | HE582009 | 0.5 |
|  |  | R: | TCCTTGATATCCCCTTCACA | |  |  |  |  |
| Am-3mer-16 | (ACA)_8_ | F: | TGGATCACGCTGCAACAAC | | 336 | 58 | HE582010 | 0.7 |
|  |  | R: | ATGGGGGAGAATGCCCCACG | |  |  |  |  |
| Am-3mer-71A | (AAACA)_5_ | F: | TACGGGTTGCTTAGCCTGC | | 198 | 60 | HE582011 | 0.2 |
|  |  | R: | TGTTAGCTCAGTCTCTTCCGT | |  |  |  |  |
| Am-3mer-71B | (CAG)_2_(CAA)_8_ | F: | CTACGGAAGAGACTGAGCT | | 204 | 60 | HE582012 | 0.4 |
|  |  | R: | GATGAGTTACCAATCCCGGT | |  |  |  |  |
| Am-3mer-114 | (TGG)_5_(AGG)_5_ | F: | ATTGCCTAGGGGTGTTCAC | | 264 | 52 | HE582013 | 0.6 |
|  |  | R: | GCTACTTGGACGCTCTTGG | |  |  |  |  |
| Am-3mer-117 | (GT)_20_ | F: | CACAGAAACCCACTGTGAC | | 248 | 52 | HE582014 | 0.7 |
|  |  | R: | GCACACTATCGCAAAAGGCAC | |  |  |  |  |
| Am-3mer-118 | (CAA)_15_ | F: | ACTACAAACCGTTGCATGA | | 306 | 55 | HE582015 | 0.7 |
|  |  | R: | ATTATATGGAGGGGGTACGT | |  |  |  |  |
| Am-3mer-124 | (TGT)_18_ | F: | GCCTTGATGAGGTTGACCT | | 207 | 60 | HE582016 | 0.4 |
|  |  | R: | GAGATTGAAGGACATCCACA | |  |  |  |  |
| Am-3mer-143 | (GTT)_17_ | F: | GGGGATTAGAAGAAGCGGCA | | 227 | 52 | HE582017 | 0.4 |
|  |  | R: | TCAACAGGCTAACCAATGAC | |  |  |  |  |
| Am-3mer-197 | (TGT)_18_ | F: | CAGCCTATTGTCTTGAGGAGG | | 285 | 55 | HE582018 | 0.6 |
|  |  | R: | GGACACCCTACAAACCGTTGC | |  |  |  |  |
| Am-3mer-239 | (CAA)_19_ | F: | GGACATTCCAAAAATCGCTGT | | 281 | 54 | HE582019 | 0.8 |
|  |  | R: | CCATGGTTGGGTTGACCTTGG | |  |  |  |  |

Supplementary Table S2 (Continued). Repeat motifs, primer sequence, allelic size, optimized annealing temperature (*Ta*) and PIC at 15 polymorphic microsatellite loci from *A. formosana*.

| Locus | Repeat motif |  | Primer sequence (5' - 3' ) | Allelic size (bp) | *Ta* ( ℃) | GenBank accession no. | PIC |
| --- | --- | --- | --- | --- | --- | --- | --- |
| Am-2mer-1-60 | (CA)_25_(CT)_44_ | F: | CCTCCTTTCCATAGAAAACG | 256 | 55 | HE582020 | 0.8 |
|  |  | R: | TCCTATCCATGTTTGGCTCC |  |  |  |  |
| Am-2mer-1-96 | (GA)_66_(GT)_12_ | F: | GGTGTATTAGAAGGCTGAGG | 276 | 55 | HE582021 | 0.9 |
|  |  | R: | CATGAGATGGTCTTCATTGG |  |  |  |  |
| Am-2mer-7-9 | (TC)_38_ | F: | TCCTTTAAGAGTGACACCTC | 231 | 59 | HE582022 | 0.8 |
|  |  | R: | TGACCCGAGGGTGAGGAATG |  |  |  |  |

Supplementary Table S3. Mean LnP(K) and ΔK for clusters using Bayesian assignment test in STRUCTURE to infer the number of clusters K in *A. formosana*.

|  | Total | |  | CHA | |  | DAWU | |  | DL | |
| --- | --- | --- | --- | --- | --- | --- | --- | --- | --- | --- | --- |
| K | Mean LnP(K) | ΔK |  | Mean LnP(K) | ΔK |  | Mean LnP(K) | ΔK |  | Mean LnP(K) | ΔK |
| 1 | -26120.8 | - |  | -13428.8 | - |  | -7607.2 | - |  | -1993.5 | - |
| 2 | -23506.4 | 5096.0 |  | -12832.5 | 129.4 |  | -6812.5 | 5987.3 |  | -1834.1 | 183.5 |
| 3 | -22790.4 | 0.5 |  | -12441.1 | 89.3 |  | -6522.8 | 302.0 |  | -1737.1 | 50.4 |
| 4 | -22002.1 | 294.1 |  | -12148.3 | 53.0 |  | -6330.7 | 0.4 |  | -1657.9 | 104.6 |
| 5 | -21616.5 | 12.3 |  | -11901.7 | 45.2 |  | -6133.5 | 1.7 |  | -1595.8 | 0.9 |
| 6 | -21321.6 | 0.1 |  | -11715.5 | 0.3 |  | -5997.4 | 0.3 |  | -1531.9 | 133.6 |
| 7 | -21018.8 | 0.5 |  | -11527.5 | 14.5 |  | -5841.5 | 1.8 |  | -1499.1 | 5.4 |
| 8 | -20744.6 | 1.8 |  | -11376.1 | 4.3 |  | -5726.1 | 13.1 |  | -1474.0 | 1.5 |
| 9 | -20566.2 | 0.2 |  | -11245.5 | 1.5 |  | -5645.5 | 0.8 |  | -1446.6 | 37.0 |
| 10 | -20373.6 | 0.4 |  | -11105.5 | 0.2 |  | -5555.1 | 14.2 |  | -1442.7 | 1.0 |
| 11 | -20225.5 | 0.7 |  | -10961.1 | 3.1 |  | -5523.5 | 1.3 |  | -1463.8 | 4.6 |
| 12 | -20028.8 | 0.1 |  | -10906.4 | 2.1 |  | -5442.9 | 0.6 |  | -1464.4 | 2.5 |
| 13 | -19830.8 | 3.0 |  | -10893.8 | 2.6 |  | -5372.8 | 2.3 |  | -1478.4 | 2.4 |
| 14 | -19704.3 | 0.5 |  | -10774.6 | 0.6 |  | -5332.4 | 0.2 |  | -1520.4 | 1.3 |
| 15 | -19613.8 | 2.0 |  | -10701.6 | 0.7 |  | -5300.4 | 1.4 |  | -1543.3 | 0.1 |
| 16 | -19388.9 | 1.4 |  | -10616.1 | 0.4 |  | -5231.1 | 9.1 |  | -1569.1 | 0.4 |
| 17 | -19240.0 | 0.4 |  | -10517.9 | 0.1 |  | -5182.5 | 2.4 |  | -1586.9 | 1.1 |
| 18 | -19110.2 | 0.6 |  | -10425.9 | 0.7 |  | -5147.5 | 0.7 |  | -1621.3 | 1.5 |
| 19 | -18997.3 | 0.8 |  | -10335.3 | 2.9 |  | -5116.2 | 0.4 |  | -1623.9 | 2.6 |
| 20 | -18867.1 | - |  | -10263.3 | - |  | -5084.4 | - |  | -1659.7 | - |

Note: Values in bold type indicate the high proportion of membership for each population that we used in this study.

Supplementary Table S4. Mean LnP(K), ΔK and DIC (deviance information criterion) for clusters using Bayesian assignment test in InStruct to infer the number of clusters K in *A. formosana*.

|  | Total | | |  | CHA | | |  | DAWU | | |  | DL | | |
| --- | --- | --- | --- | --- | --- | --- | --- | --- | --- | --- | --- | --- | --- | --- | --- |
| K | Mean LnP(K) | ΔK | DIC |  | Mean LnP(K) | ΔK | DIC |  | Mean LnP(K) | ΔK | DIC |  | Mean LnP(K) | ΔK | DIC |
| 1 | -20539.2 | - | 41078.4 |  | -10837.5 | - | 21675.0 |  | -6090.6 | - | 12181.2 |  | -1626.2 | - | 3252.3 |
| 2 | -18643.5 | 0.2 | 37287.1 |  | -10363.0 | 0.1 | 20726.0 |  | -5481.8 | 2.3 | 10963.7 |  | -1499.2 | 0.7 | 2998.4 |
| 3 | -18008.4 | 0.04 | 36016.9 |  | -9988.8 | 0.0 | 19977.5 |  | -5198.3 | 0.2 | 10396.7 |  | -1406.9 | 0.1 | 2813.8 |
| 4 | -18008.4 | 0.03 | 34797.3 |  | -9644.3 | 0.0 | 19288.6 |  | -5008.5 | 0.0 | 10017.1 |  | -1365.1 | 0.0 | 2730.3 |
| 5 | -16943.4 | 0.0 | 33886.8 |  | -9353.3 | 0.0 | 18706.5 |  | -4842.4 | 0.0 | 9684.7 |  | -1327.5 | 0.0 | 2654.9 |
| 6 | -16470.4 | 0.0 | 32940.7 |  | -9113.0 | 0.0 | 18226.0 |  | -4739.8 | 0.0 | 9479.5 |  | -1295.5 | 0.04 | 2590.9 |
| 7 | -16059.6 | 0.0 | 32119.2 |  | -8876.9 | 0.0 | 17753.8 |  | -4663.4 | 0.0 | 9326.8 |  | -1287.3 | 0.0 | 2574.6 |
| 8 | -15729.0 | 0.0 | 31458.0 |  | -8704.8 | 0.0 | 17409.7 |  | -4585.8 | 0.0 | 9171.6 |  | **-**1286.6 | 0.0 | 2573.2 |
| 9 | -15482.5 | 0.0 | 30964.9 |  | -8549.3 | 0.0 | 17098.6 |  | -4530.8 | 0.0 | 9061.6 |  | -1288.2 | 0.0 | 2576.4 |
| 10 | -15248. | 0.0 | 30496.5 |  | -8443.9 | 0.0 | 16887.8 |  | -4476.2 | 0.0 | 8952.5 |  | -1289.2 | 0.0 | 2578.4 |
| 11 | -15014.3 | 0.0 | 30028.7 |  | -8365.4 | 0.0 | 16730.7 |  | -4435.5 | 0.0 | 8870.9 |  | -1294.3 | 0.0 | 2588.6 |
| 12 | -14811.2 | 0.0 | 29622.4 |  | -8296.0 | 0.0 | 16591.9 |  | -4414.0 | 0.0 | 8828.0 |  | -1299.6 | 0.0 | 2599.2 |
| 13 | -14621.5 | 0.0 | 29243.0 |  | -8231.8 | 0.0 | 16463.5 |  | -4411.7 | 0.0 | 8823.4 |  | -1301.9 | 0.0 | 2603.7 |
| 14 | -14460.1 | 0.0 | 28920.1 |  | -8190.5 | 0.0 | 16380.9 |  | -4418.1 | 0.0 | 8836.1 |  | -1305.5 | 0.0 | 2611.0 |
| 15 | -14315.3 | 0.0 | 28630.5 |  | -8155.0 | 0.0 | 16310.1 |  | -4426.3 | 0.0 | 8852.6 |  | -1310.6 | 0.0 | 2621.3 |
| 16 | -14208.4 | 0.0 | 28416.8 |  | -8131.2 | 0.0 | 16262.3 |  | -4437.3 | 0.0 | 8874.5 |  | -1312.9 | 0.0 | 2625.7 |
| 17 | -14108.4 | 0.0 | 28216.8 |  | -8114.2 | 0.0 | 16228.3 |  | -4448.7 | 0.0 | 8897.5 |  | -1319.0 | 0.0 | 2637.9 |
| 18 | -14043.9 | 0.0 | 28087.8 |  | -8106.9 | 0.0 | 16213.8 |  | -4463.7 | 0.0 | 8927.4 |  | -1325.2 | 0.0 | 2650.3 |
| 19 | -13996.3 | 0.0 | 27992.5 |  | -8123.1 | 0.0 | 16246.3 |  | -4474.0 | 0.0 | 8948.0 |  | -1328.5 | 0.0 | 2657.0 |
| 20 | -13949.7 | - | 27899.4 |  | -8138.3 | - | 16276.7 |  | -4486.8 | - | 8973.5 |  | -1335.1 | - | 2670.2 |

Note: Values in bold type indicate the high proportion of membership for each population and we used in this study

Supplementary Table S5. Estimates the six IMa model parameters in *A. formosana*, The N, M and T are effective population size (*q*), migration rate (*m*) and divergence time (*t*) scaled by the mutation rate. The N_1_ is the effective population size in the CHA population, N_2_ is the effective population size in DAWU & DL population, N_A_ is the effective population size ancestral, M_1_**_→_**_2_ is the migration rate from CHA to DAWU & DL, M_2_**_→_**_1_ is the migration rate from DAWU & DL to CHA and the T is divergence time. The mutation rate was referred to *Thuja plicata* Donn mutation rate for microsatellite was 6.3×10^-4^ (95% confidence interval of 3.0×10^-5^ - 4.0×10^-3^) mutations per locus per generation (O’Connell and Ritland 2004).

| CHA **vs**.  DAWU & DL | N_1_ | N_2_ | N_A_ | M_1_**_→_**_2_ | M_2_**_→_**_1_ | T |
| --- | --- | --- | --- | --- | --- | --- |
| raw data |  |  |  |  |  |  |
| HiPt | 0.1 | 0.0 | 40.7 | 0.7 | 0.1 | 0.0 |
| 95%HPDLo | 0.0 | 0.0 | 32.3 | 0.2 | 0.9 | 0.0 |
| 95%HPDHi | 0.6 | 2.9 | 43.5 | 155.3 | 205.6 | 0.6^-03^ |
| 6.3×10^-4^ |  |  |  |  |  |  |
| HiPt | 27.8 | 9.8 | 16145.2 | 0.5^-03^ | 0.1^-03^ | 0.0 |
| 95%HPDLo | 9.3 | 4.2 | 12804.8 | 0.2^-03^ | 0.6^-03^ | 0.0 |
| 95%HPDHi | 250.5 | 1131.9 | 17258.7 | 0.1 | 0.1 | 1.0 |
| μ＝3.0×10-5 |  |  |  |  |  |  |
| HiPt | 584.2 | 205.8 | 339049.2 | 0.2^-04^ | 0.4^-05^ | 0.0 |
| 95%HPDLo | 195.0 | 88.3 | 268901.7 | 0.7^-05^ | 0.3^-04^ | 0.0 |
| 95%HPDHi | 5260.8 | 23769.2 | 362432.5 | 0.5^-02^ | 0.6^-02^ | 20.0 |
| μ＝4.0×10^-3^ |  |  |  |  |  |  |
| HiPt | 4.4 | 1.5 | 2542.9 | 0.3^-02^ | 0.1^-02^ | 0.0 |
| 95%HPDLo | 1.5 | 0.7 | 2016.8 | 0.1^-02^ | 0.4^-02^ | 0..0 |
| 95%HPDHi | 39.5 | 178.3 | 2718.2 | 0.6 | 0.8 | 0.2 |

Note: (1) HiPt: The value of the bin with the highest count.

(2) 95%HPDLo: The lower bound of the estimated 95% highest posterior density (HPD) interval.

(3) 95%HPDHi: The upper bound of the estimated 95% highest posterior density (HPD) interval.

Supplementary Table S6. Results of estimates of the effective population size (*Ne*) via LDNe analysis in *A. formosana*, independent comparisons, overall *r^2^*^,^ expected *r^2^* and percentage of 95% confidence intervals are included in the analysis.

| Lowest Alleles Frequency used = 0.05 | | | | |
| --- | --- | --- | --- | --- |
|  | Independent Comparisons | Overall *r^2^* | Expected *r^2^* | *Ne* (95% CIs for *Ne*) |
| Total | 1685 | 0.1^-01^ | 0.2^-02^ | 39.7 (36.4-43.2)^1^ (30.8-50.3)^2^ |
| CHA | 1388 | 0.1^-01^ | 0.3^-02^ | 94.4 (81.9-109.1)^1^ (74.4-121.0)^2^ |
| DAWU | 966 | 0.2^-01^ | 0.4^-02^ | 23.4 (20.4-26.6)^1^ (16.3-32.7)^2^ |
| DL | 721 | 0.3^-01^ | 0.2^-01^ | 33.0 (24.8-45.2)^1^ (22.7-50.8)^2^ |
| Lowest Alleles Frequency used = 0.02 | | | | |
|  | Independent Comparisons | Overall *r^2^* | Expected *r^2^* | *Ne* (95% CIs for *Ne*) |
| Total | 2591 | 0.1^-01^ | 0.2^-02^ | 49.1 (45.7-52.6)^1^ (39.8-59.9)^2^ |
| CHA | 2109 | 0.1^-01^ | 0.3^-02^ | 102.1 (90.7-115.2)^1^ (78.7-134.2)^2^ |
| DAWU | 1381 | 0.2^-01^ | 0.4^-02^ | 28.7 (25.5-32.1)^1^ (20.4- 39.6)^2^ |
| DL | 1085 | 0.2^-01^ | 0.2^-01^ | 37.9 (29.6-50.0)^1^ (25.5-60.8)^2^ |
| Lowest Alleles Frequency used = 0.01 | | | | |
|  | Independent Comparisons | Overall *r^2^* | Expected *r^2^* | *Ne* (95% CIs for *Ne*) |
| Total | 3024 | 0.1^-01^ | 0.2^-02^ | 54.7 (51.1-58.4)^1^ (44.3-66.8)^2^ |
| CHA | 2258 | 0.1^-01^ | 0.3^-02^ | 105.1 (93.6-118.3)^1^ (81.8-136.9)^2^ |
| DAWU | 1692 | 0.2^-01^ | 0.4^-02^ | 36.5 (32.8-40.7)^1^ (26.8-49.6)^2^ |
| DL | 1131 | 0.2^-01^ | 0.2^-01^ | 45.1 (34.6-61.2)^1^ (29.4-77.1)^2^ |

1. Parametric

2. JackKnife on Loci

Supplementary Table S7. Estimates of recent migration rates (including 95% confidence intervals) between 3 populations (CHA, DAWU and DL) of *A. formosana* derived from the program BAYESASS.

| Migration into | Migration from | | |
| --- | --- | --- | --- |
|  | CHA | DAWU | DL |
| CHA | **1.0**  (1.0-1.0) | 0.5^-03^  (6.3^-06^-0.2^-02^) | 0.6^-03^  (4.3^-06^-0.3^-02^) |
| DAWU | 0.1^-02^  (4.9^-05^-0.5^-02^) | **0.8**  (7.8^-01^-0.8) | 0.2  (1.7^-01^-2.2^-01^) |
| DL | 0.3^-02^  (2.1^-05^-0.1^-01^) | 0.3^-02^  (2.6^-05^-0.1^-01^) | **1.0**  (9.8^-01^-1.0) |

Data presented the means of proportion distributions of migrants per generation for each population of individuals. The populations from which each individual are listed in the rows, while the populations from which they migrated are listed in the columns. Values in bold are the proportions of individuals derived from each generation's source populations. Values in parentheses below migration rates are 95% confidence intervals.

Supplementary Table S8. The detailed dissection of the STRUCTURE and GENELAND individual genetic structure analysis results for CHA, DAWU, and DL populations of *A. formosana*. Each component of the clusters is examined layer by layer. The percentage representation of samples under each stratified clustering is also presented, giving in-depth insights into the genetic structure. Additionally, the selection of ex-situ conservation trees for each Management Unit is displayed, which includes information on the Diameter at Breast Height (DBH) and health grading standards (H).

| Population | STRUCTURE | | GENELAND  (Management Units, MUs) | Ex-situ conservation trees |
| --- | --- | --- | --- | --- |
| CHA | K=2 | K=3 | K=8 |  |
|  | CS-2-1 (37.3%) | CS-3-1 (9.8%) | CG-8-1 (21.4%) |  |
|  |  |  | CG-8-3 (42.9%) |  |
|  |  |  | CG-8-6 (28.6%) | 366 (DBH=6.0, H=4) |
|  |  |  | CG-8-7 (7.1%) |  |
|  |  | CS-3-2 (5.6%) | CG-8-1 (12.5%) |  |
|  |  |  | CG-8-3 (62.5%) | 165 (DBH=9.0, H=4) |
|  |  |  | CG-8-6 (25.0%) |  |
|  |  | CS-3-3 (84.6%) | CG-8-1 (1.7%) |  |
|  |  |  | CG-8-2 (0.8%) | 477 (DBH=20.0, H=5) |
|  |  |  | CG-8-3 (14.0%) |  |
|  |  |  | CG-8-5 (2.5%) |  |
|  |  |  | CG-8-6 (22.3%) | 362 (DBH=15.0, H=5) |
|  |  |  | CG-8-7 (57.0%) | 253 (DBH=9.0, H=4)  748 (DBH=7.0, H=4)  749 (DBH=8.0, H=4)  795 (DBH=14.0, H=5)  106 (DBH=20.0, H=4)  44 (DBH=28, H=4) |
|  |  |  | CG-8-8 (1.7%) | 163 (DBH=6.0, H=4) |
|  | CS-2-2 (62.7%) | CS-3-1 (55.9%) | CG-8-1 (36.1%) | 779 (DBH=14.0, H=4)  767 (DBH=8.0, H=4)  736 (DBH=17, H=4)  742 (DBH=12, H=4) |
|  |  |  | CG-8-2 (11.3%) | 553 (DBH=4.0, H=4)  590 (DBH=4.0, H=4)  652 (DBH=14.0, H=3) |
|  |  |  | CG-8-3 (26.3%) | 299 (DBH=9.0, H=4)  311 (DBH=8.0, H=4)  404 (DBH=16.0, H=4)  320 (DBH=15.0, H=5)  289 (DBH=22, H=4) |
|  |  |  | CG-8-4 (6.0%) |  |
|  |  |  | CG-8-5 (2.3%) |  |
|  |  |  | CG-8-6 (16.5%) | 368 (DBH=9.0, H=4)  186 (DBH=13.0, H=5) |
|  |  |  | CG-8-7 (0.8%) |  |
|  |  |  | CG-8-8 (0.8%) |  |
|  |  | CS-3-2 (40.3%) | CG-8-1 (4.0%) | 759 (DBH=8.0, H=4) |
|  |  |  | CG-8-2 (6.0%) |  |
|  |  |  | CG-8-3 (24.0%) |  |
|  |  |  | CG-8-4 (56.0%) | 439 (DBH=6.0, H=5)  677 (DBH=9.0, H=4)  596 (DBH=20.0, H=4)  551 (DBH=22.0, H=4) |
|  |  |  | CG-8-5 (9.0%) | 698 (DBH=3.0, H=4)  722 (DBH=8.0, H=4)  708 (DBH=22.0, H=4)  712 (DBH=11.0, H=4)  717 (DBH=11.0, H=3) |
|  |  |  | CG-8-7 (1.0%) |  |
|  |  | CS-3-3 (3.8%) | CG-8-7 (85.7%) |  |
|  |  |  | CG-8-8 (14.3%) |  |
| DAWU | K=2 | K=3 | K=15 |  |
|  | DS-2-1 (57.1%) | DS-3-1 (56.8%) | DG-15-1 (2.8%) |  |
|  |  |  | DG-15-3 (5.6%) | 104 (DBH=2.8) |
|  |  |  | DG-15-4 (9.9%) | 107 (DBH=2.5) |
|  |  |  | DG-15-5 (7.0%) | 210 (DBH=2.8) |
|  |  |  | DG-15-7 (14.1%) |  |
|  |  |  | DG-15-8 (9.9%) | 200 (DBH=3.5)  206 (DBH=7.0) |
|  |  |  | DG-15-9 (28.2%) | 220 (DBH=30.0)  224 (DBH=9.5)  225 (DBH=21.5) |
|  |  |  | DG-15-10 (8.5%) | 141 (DBH=5.7)  143 (DBH=3.0) |
|  |  |  | DG-15-12 (1.4%) |  |
|  |  |  | DG-15-13 (12.7%) |  |
|  |  | DS-3-3 (43.2%) | DG-15-3 (5.6%) | 172 (DBH=3.0) |
|  |  |  | DG-15-4 (1.9%) | 80 (DBH=2.5)  81 (DBH=3.0) |
|  |  |  | DG-15-5 (5.6%) | 184 (DBH=2.5)  185 (DBH=3.5) |
|  |  |  | DG-15-7 (25.9%) | 18 (DBH=11.0) |
|  |  |  | DG-15-9 (1.9%) | 221 (DBH=26.7) |
|  |  |  | DG-15-10 (1.9%) |  |
|  |  |  | DG-15-11 (25.9%) | 5 (DBH=10.0)  27 (DBH=3.0)  35 (DBH=11.0) |
|  |  |  | DG-15-12 (20.4%) |  |
|  |  |  | DG-15-14 (1.9%) |  |
|  |  |  | DG-15-15 (9.3%) |  |
|  | DS-2-2 (42.9%) | DS-3-2 (94.7%) | DG-15-1 (15.7%) | 133 (DBH=24.5)  173 (DBH=11.5)  174 (DBH=27.0)  178 (DBH=12.5) |
|  |  |  | DG-15-2 (10.1%) | 114 (DBH=19.0)  115 (DBH=27.0)  119 (DBH=25.0)  120 (DBH=17.0) |
|  |  |  | DG-15-6 (12.4%) | 211 (DBH=11.0)  212 (DBH=13.0)  214 (DBH=11.0)  215 (DBH=28.0)  217 (DBH=18.0) |
|  |  |  | DG-15-7 (13.5%) | 97 (DBH=14.2)  118 (DBH=17.5)  123 (DBH=34.0)  131 (DBH=25.0)  160 (DBH=11.5) |
|  |  |  | DG-15-11 (1.1%) | 25 (DBH=15.7) |
|  |  |  | DG-15-12 (11.2%) | 56 (DBH=8.2)  58 (DBH=9.0)  73 (DBH=7.5)  111 (DBH=23.8) |
|  |  |  | DG-15-13 (12.4%) | 83 (DBH=14.0)  88 (DBH=20.0)  95 (DBH=11.5)  96 (DBH=12.9)  125 (DBH=16.0) |
|  |  |  | DG-15-14 (5.6%) | 9 (DBH=16.5)  16 (DBH=12.5)  17 (DBH=14.0)  20 (DBH=17.0)  109 (DBH=14.7) |
|  |  |  | DG-15-15 (18.0%) | 30 (DBH=16.5)  36 (DBH=22.0)  163 (DBH=13.0)  164 (DBH=28.0)  165 (DBH=12.5)  168 (DBH=9.0) |
|  |  | DS-3-3 (5.3%) | DG-15-6 (20.0%) |  |
|  |  |  | DG-15-11 (20.0%) |  |
|  |  |  | DG-15-12 (60.0%) |  |
| DL | K=2 | K=6 | K=6 |  |
|  | LS-2-1 (70.8%) | LS-6-1 (2.2%) | LG-6-1 (100%) | C28 (DBH=14.8) |
|  |  | LS-6-2 (32.6%) | LG-6-2 (100%) | A1 (DBH=24.5)  A2 (DBH=10.2)  A7 (DBH=11.8)  A8 (DBH=11.2)  A11 (DBH=16.3)  A15 (DBH=15.8) |
|  |  | LS-6-3 (21.7%) | LG-6-2 (10.0%) |  |
|  |  |  | LG-6-3 (80.0%) | B92 (DBH=10.5)  B95 (DBH=11.7) |
|  |  |  | LG-6-5 (10.0%) |  |
|  |  | LS-6-4 (19.6%) | LG-6-4 (10.0%) | C30 (DBH=9.9)  C33 (DBH=8.2)  C29 (DBH=16.7)  C35 (DBH=26.7)  C36 (DBH=15.6) |
|  |  | LS-6-5 (23.9%) | LG-6-3 (9.1%) | B96 (DBH=16.5) |
|  |  |  | LG-6-5 (72.7%) | C11 (DBH=7.6)  C14 (DBH=9.5)  C15 (DBH=10.8)  C18 (DBH=14.0) |
|  |  |  | LG-6-6 (18.2%) |  |
|  | LS-2-2 (29.2%) | LS-6-1 (42.1%) | LG-6-1 (87.5%) | C22 (DBH=10.0)  C27 (DBH=14.3)  C26 (DBH=20.0) |
|  |  |  | LG-6-3 (12.5%) | C19 (DBH=25.0) |
|  |  | LS-6-5 (10.5%) | LG-6-1 (50.0%) |  |
|  |  |  | LG-6-6 (50.0%) |  |
|  |  | LS-6-6 (47.4%) | LG-6-2 (11.1%) |  |
|  |  |  | LG-6-6 (88.9%) | C3 (DBH=26.0)  C6 (DBH=10.8)  C7 (DBH=11.2) |


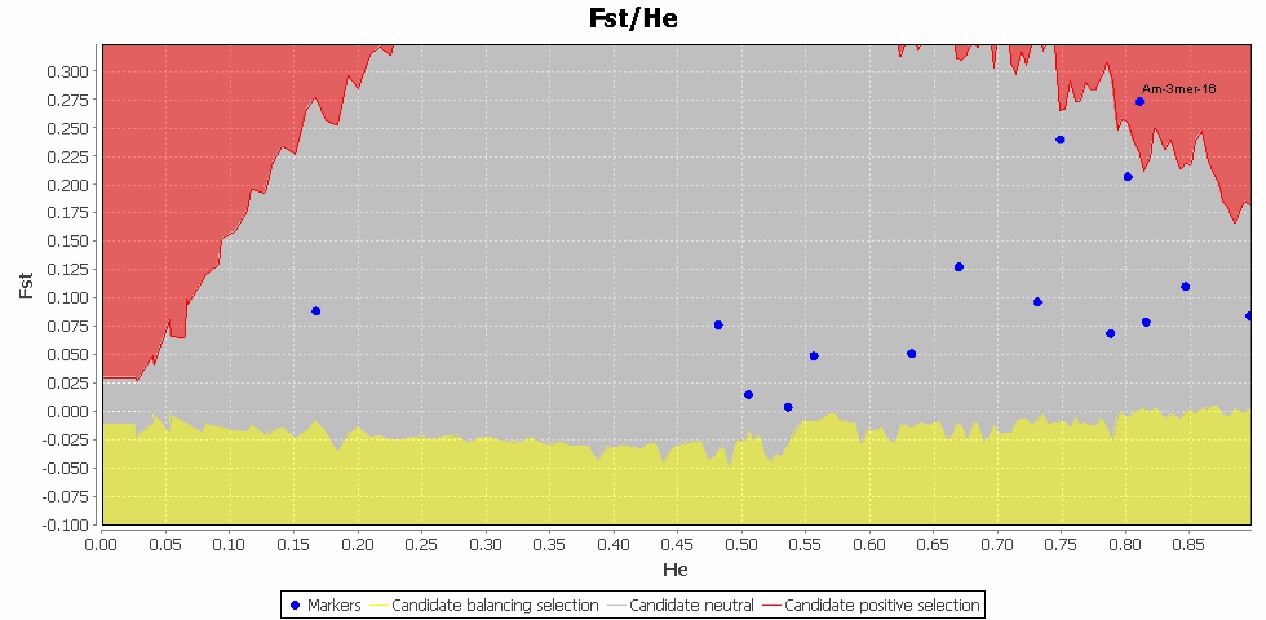


Supplementary Figure S1. Plot of *F_ST_* versus heterozygosity (*He*) to test the natural selection effect in 15 microsatellite loci using LOSITAN. There is a grey area for neutrality, and the outliers are identified as candidates for directional selection or balancing selection.


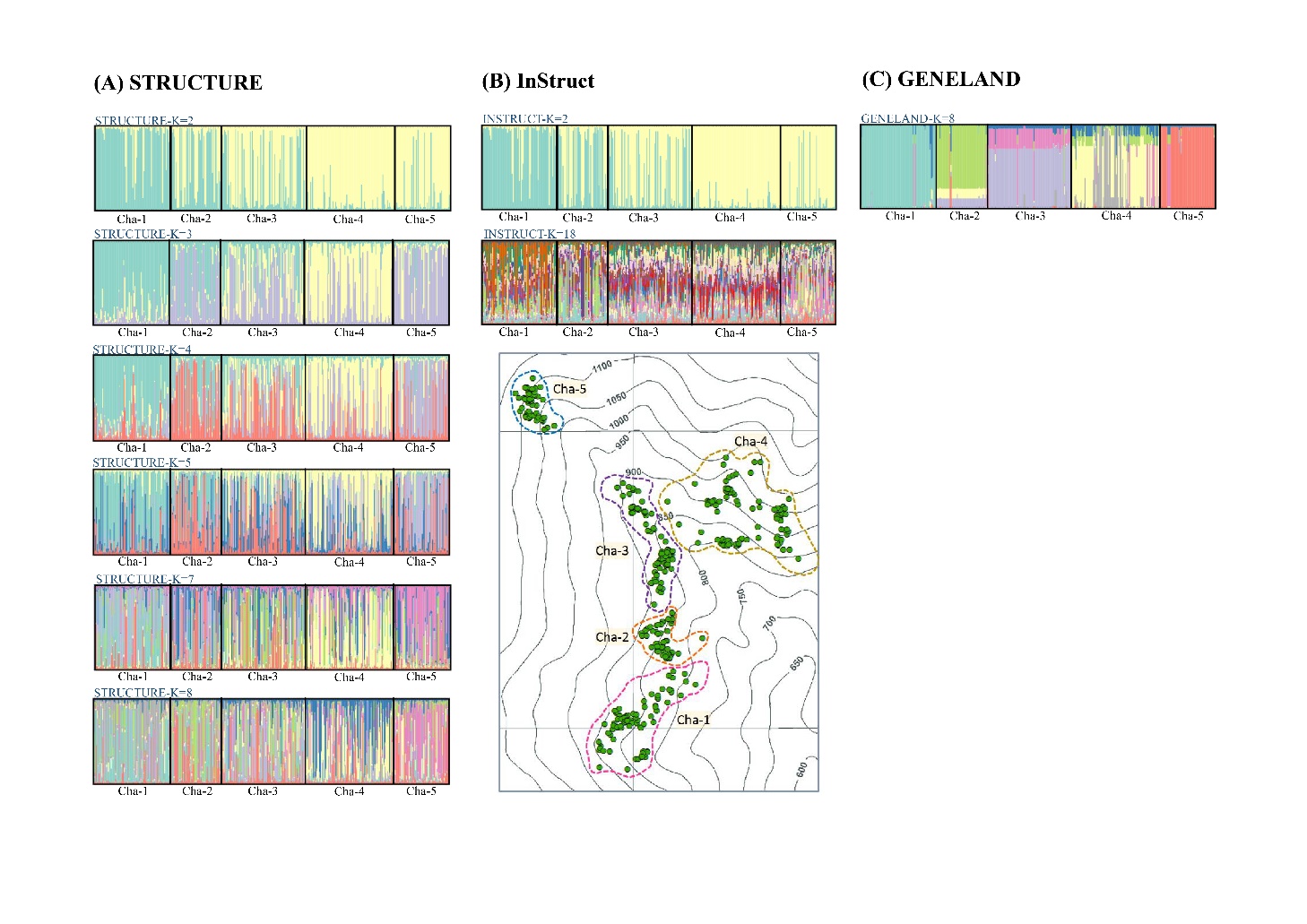


Supplementary Figure S2. The results of Bayesian clustering analysis of *A. formosana* in the CHA population using STRUCTURE (A), InStruct (B), and GENELAND (C). The best number of clusters for the STRUCTURE analysis result is K=2 (followed by K=3, 4, 5, 7, 8), the best number of clusters for the InStruc analysis result is K=18, and the best number of clusters for the GENELAND analysis result is K=8. Each color represents a different cluster, and each bar line represents a different individual.


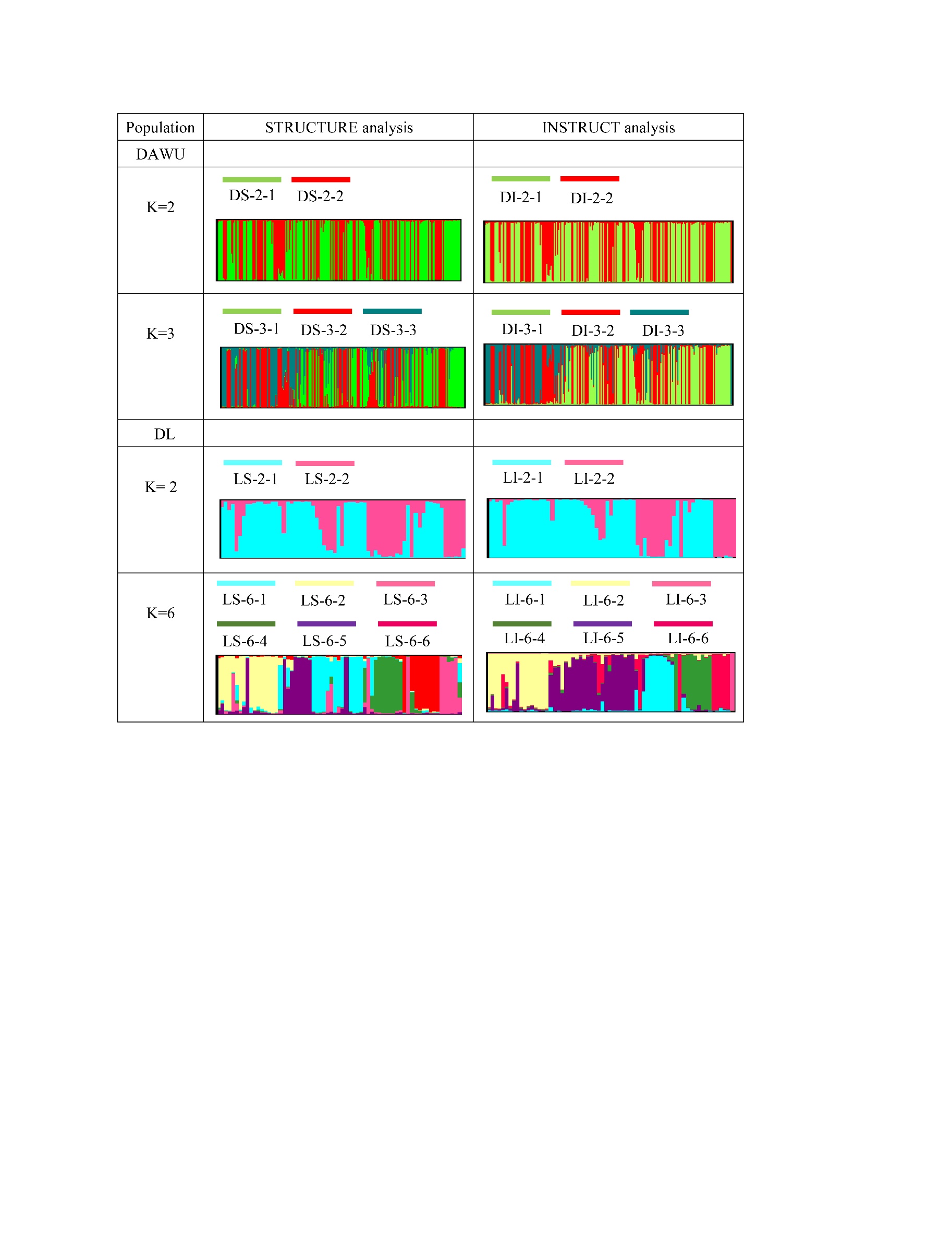


Supplementary Figure S3. The results of Bayesian clustering analysis of *A. formosana* in the DAWU and DL population using STRUCTURE, InStruct. Each color represents a different cluster, and each bar line represents a different individual. The code represented by different color blocks is shown.


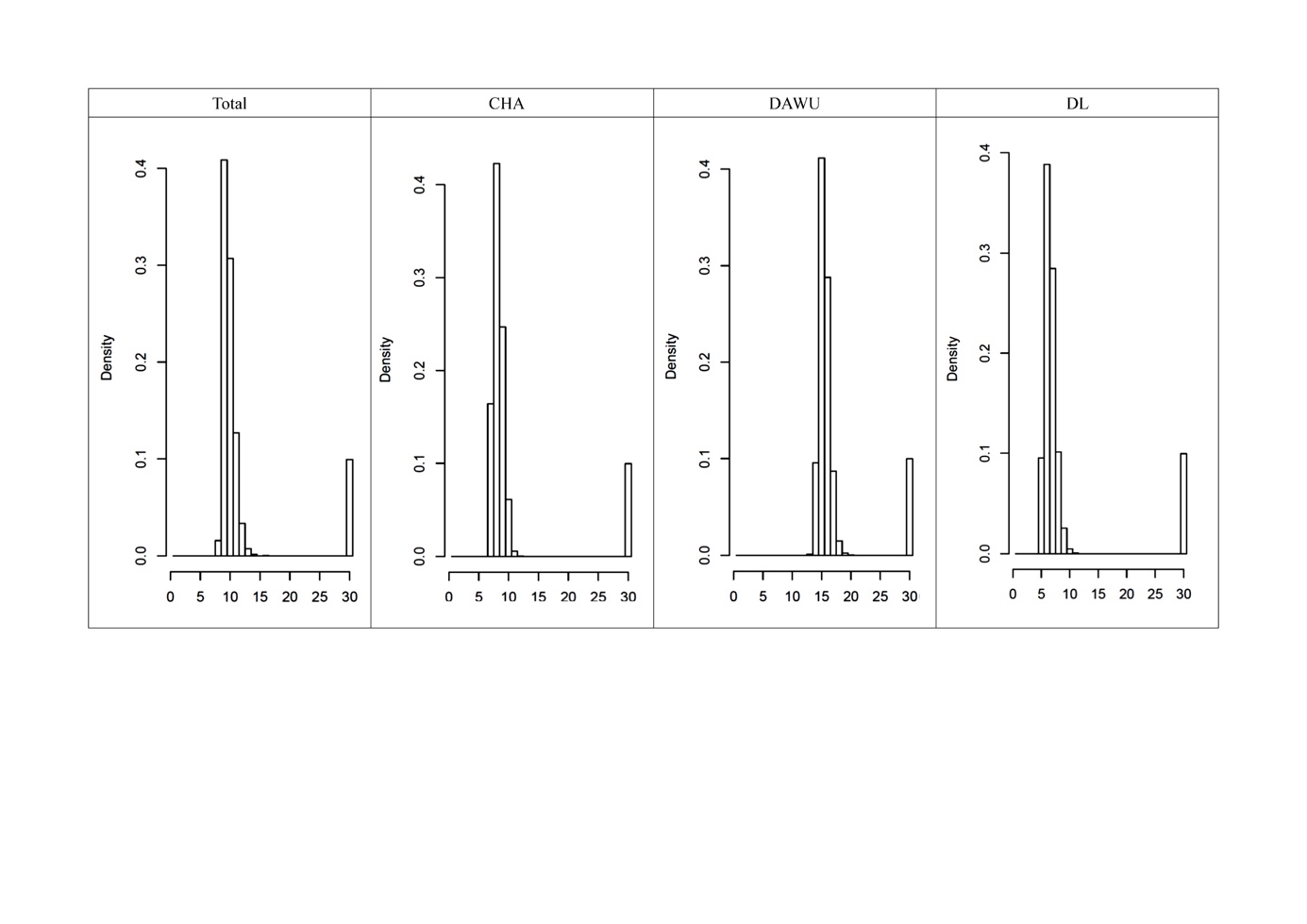


Supplementary Figure S4. Histogram from GENELAND analysis showing the average density to inferred K in total populations and three populations of CHA, DAWU and DL separate analysis results in *A. formosana*


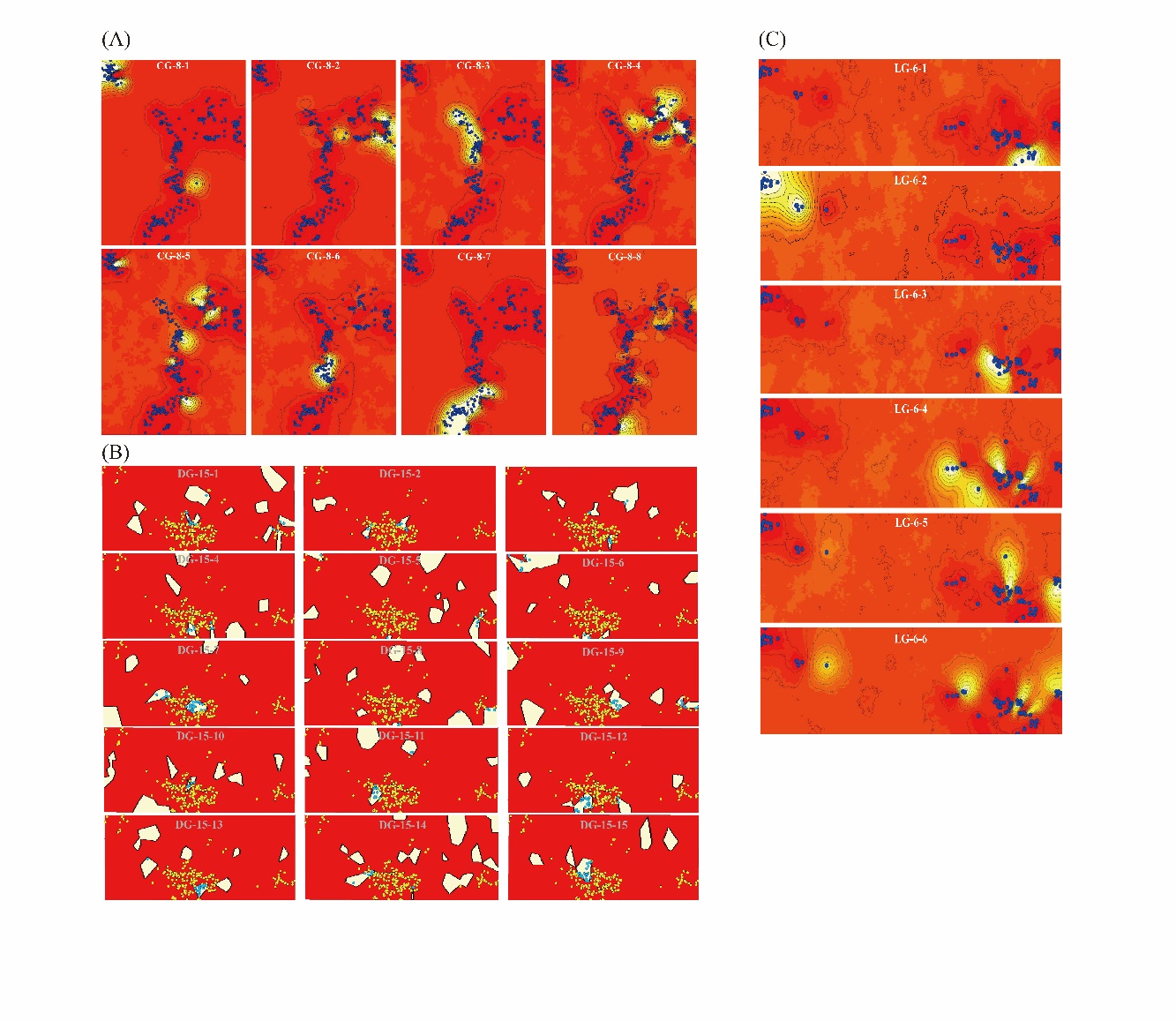


Supplementary Figure S5. Bayesian cluster analysis output maps from GENELAND using microsatellite data for the CHA (A), DAWU (B) and DL (C) populations of *A. formosana*. Maps of posterior probabilities belong to K=8, 15 and 6 for the three populations, respectively. The blue circle expressed the sampling sites. The light (white) to dark (red) color indicates the high to low probability of membership belonging to a particular subpopulation. Each plaque represents different genotypes within populations.


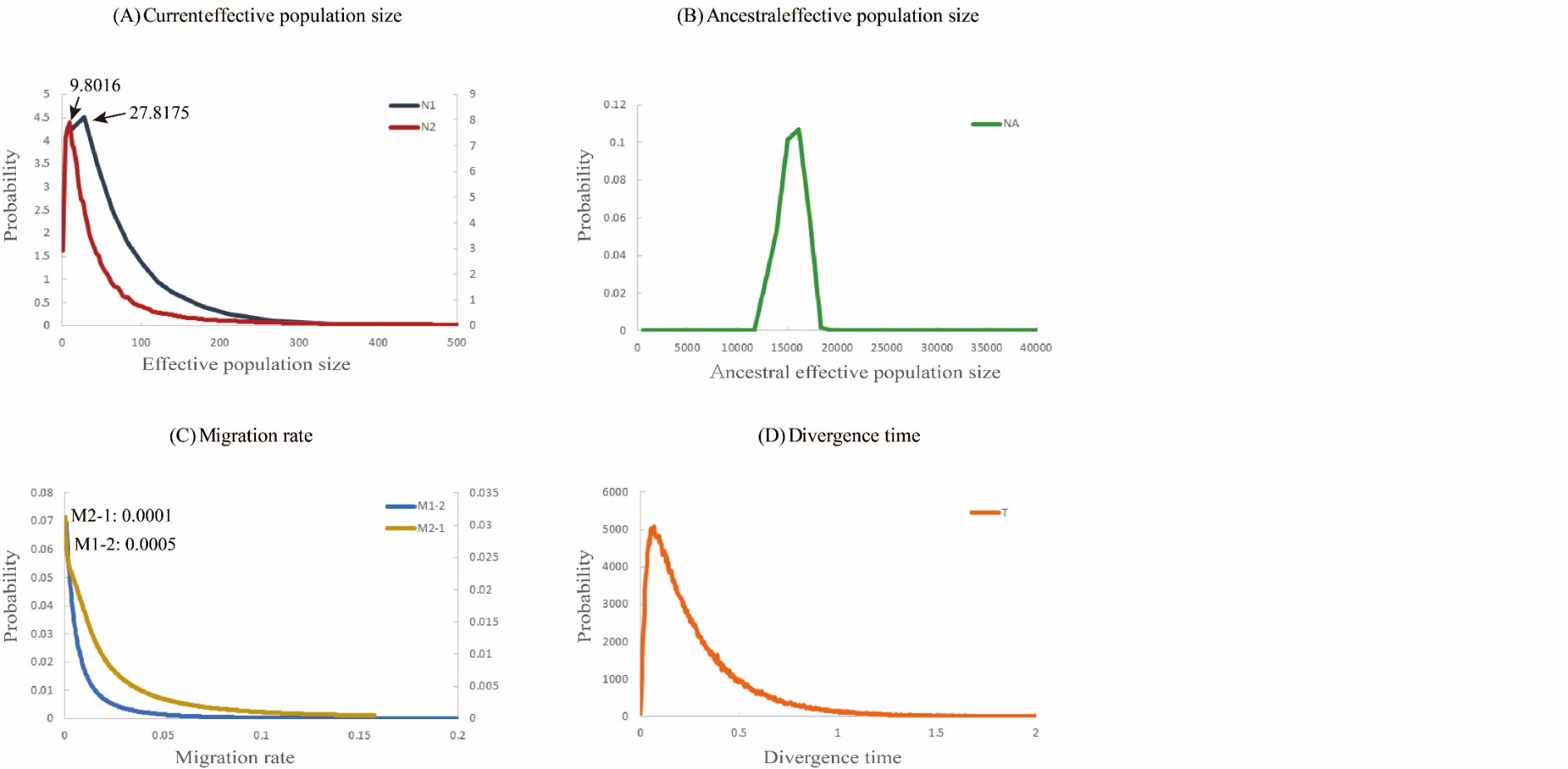


Supplementary Figure S6. The posterior distributions for (A) two current effective population sizes (N_1_ and N_2_), (B) ancestral effective population size (N_A_), (C) migration rate (M_1_**_→_**_2_ and M_2_**_→_**_1_) and (D) divergence time (T) between CHA population (population 1) and DAWU & DL population (population 2) in *A. formosana*. (scaled by the mutation rate, μ＝6.3×10^-4^ per generation)


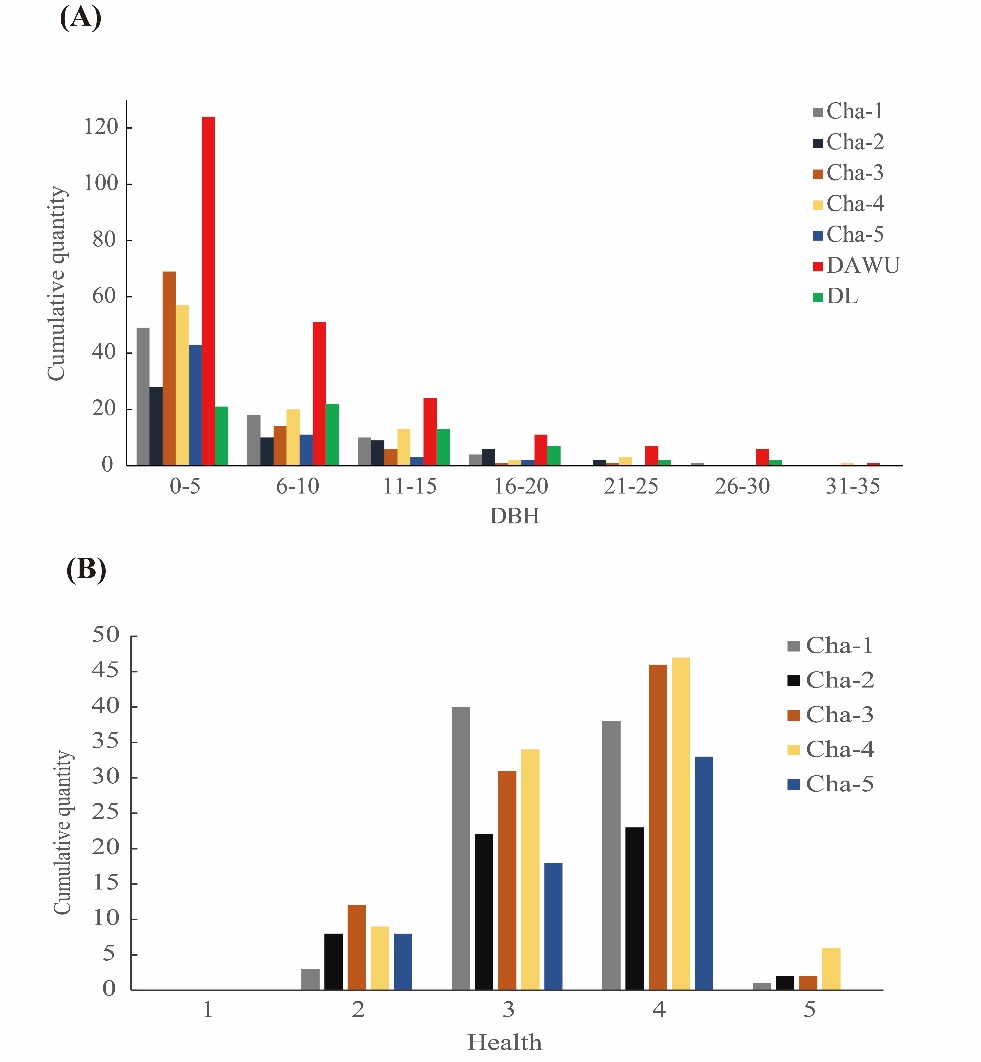


Supplementary Figure S7. Bar chart representation of the Diameter at Breast Height (DBH) (A) and Health Grading Standards (B) data for *A. formosana* samples collected from different populations in this study.
